# Long noncoding RNA RP11-757G1.5 sponges miR-139-5p and upregulates YAP1 thereby promoting the proliferation and liver, spleen metastasis of colorectal cancer

**DOI:** 10.1186/s13046-020-01717-5

**Published:** 2020-10-06

**Authors:** Xiaojian Zhu, Fanqin Bu, Ting Tan, Qilin Luo, Jinfeng Zhu, Kang Lin, Jun Huang, Chen Luo, Zhengming Zhu

**Affiliations:** 1 The Seventh Affiliated Hospital of Sun Yat-sen University, Shenzhen, China; 2 Zhongshan School of Medicine, Sun Yat-Sen University, Guangzhou, China; 3 Department of General Surgery, The Second Affiliated Hospital of Nanchang University, Nanchang, 330006 China; 4 Jiangxi Medical College of Nanchang University, Nanchang, China; 5 Jiangxi Province Key Laboratory of Molecular Medicine, Nanchang, China; 6 Otolaryngology Head and Neck Surgery, The Second Affiliated Hospital of Nanchang University, Nanchang, China

**Keywords:** RP11-757G1.5, LncRNAs, miR-139-5p, Proliferation, ceRNA, Colorectal cancer metastasis

## Abstract

**Background:**

Accumulating evidence indicates that long non-coding RNAs (lncRNAs) acting as crucial regulators in tumorigenesis. However, its biological functions of lncRNAs in colorectal cancer (CRC) have not been systematically clarified.

**Methods:**

An unbiased screening was performed to identify disregulated lncRNAs revealed to be implicated in CRC carcinogenesis according to an online-available data dataset. In situ hybridization (ISH), RT-qPCR and RNA fluorescence in situ hybridization (RNA-FISH) were applied to detect RP11-757G1.5 expression in CRC tissues and cell lines. The associations of RP11-757G1.5 with clinicopathological characteristics were analyzed. Their effects on prognosis were analyzed by the Kaplan-Meier analysis, Log-rank test, Univariate and Multivariate Cox regression analysis. The potential biological function of RP11-757G1.5 in CRC was investigated by Colony formation, Edu cell proliferation, Flow cytometry, Wound healing and Transwell assays. Bioinformatics binding site analysis, Luciferase reporter assay, Ago2 immunoprecipitation assays, RNA pull-down assay, RT-qPCR and Western blotting were utilized to demonstrate the mechanism of RP11-757G1.5 acts as a molecular sponge of miR-139-5p to regulate the expression of YAP1. Finally, we further explore the potential role of RP11-757G1.5 in CRC orthotopic xenografts in vivo.

**Results:**

We discovered a novel oncogenic lncRNA RP11-757G1.5, that was overexpressed in CRC tissues, especially in aggressive cases. Moreover, up-regulation of RP11-757G1.5 strongly correlated with poor clinical outcomes of patients with CRC. Functional analyses revealed that RP11-757G1.5 promoted cell proliferation in vitro and in vivo. Furthermore, RP11-757G1.5 stimulated cell migration and invasion in vitro and in vivo. Mechanistic studies illustrated that RP11-757G1.5 regulated the expression of YAP1 through sponging miR-139-5p and inhibiting its activity thereby promoting CRC progression and development.

**Conclusions:**

Altogether, these results reveal a novel RP11-757G1.5/miR-139-5p/YAP1 regulatory axis that participates in CRC carcinogenesis and progression.

## Background

Colorectal cancer (CRC) is the third most commonly diagnosed cancer worldwide, with approximately 1.3 million new cancer cases and over 0.6 million deaths reported each year [1]. The occurrence and development of CRC involve a series of complex changes at the genetic and epigenetic levels [2]. In terms of clinical treatment, high rates of metastasis, cancer recurrence, and chemoresistance, make CRC challenging to treat at any stage. As previous studies have described that resistin exposure can block cells in the G1 phase, thereby prolonging their resistance and delaying the progression of p53 non-functional colon cancer cells [3]. In the field of molecular targeted therapy, anti-epidermal growth factor receptor monoclonal antibodies, such as cetuximab or panitumumab, combined with chemotherapy have made some progress for the treatment of patients with RAS and BRAF wild-type metastatic CRC [4]. Immunotherapy has also made great progress in the therapy of CRC, two kinds of programmed cell death 1 (PD-1) blocking antibodies, pembrolizumab and nivolumab, show efficacy in patients with metastatic CRC who have defects in repair of mismatch repair and high microsatellite instability [5, 6]. But it remains inadequate to ameliorate the clinical outcomes and prognosis of colorectal cancer patients, thus, this highlights the need for the development and further necessitating new therapeutic strategies.

Recently, lncRNAs (long non-coding RNAs) have been obseverved in a number of studies to be a key player in the incidence and growth of colorectal cancer. LncRNAs, which are also known as non-protein coding RNA molecules, consist of more than 200 nucleotides. They are said to control a wide range of functions which include post-transcriptional and chromatin modification. These type of RNAs are linked as well to the advancement of numerous cancers not excluding liver [7], colorectal [8], gastric [9], and small cell lung cancers [10]. MicroRNAs (miRNAs), a collection of non-coding RNAs, regulate a few stages of tumor genesis and cancer development and gene expression [11, 12]. Aside from this, a number of human diseases are also closely linked to the atypical expression of miRNAs; especially in cancer cases. For example, Anti-miR-203, by acting on SOCS3, will hinder breast cancer evolution and stemness [13]. Previous studies have exhibited that the suppression of miR-203 can inhibit tumor growth and cell proliferation in ER-positive breast carcinegenic cells by inhibiting Cyclin D1 and pStat3. In ovarian cancer cases, the upregulation of MiR-205 was observed and the overexpression of miR-205 was associated with tumor metastasis [14]. Suggestions made by recent studies show that the modulation of gene expression by some IncRNAs is carried out through the suppression of miRNA levels. Precisely, lncRNAs act as competitive endogenous RNAs (ceRNA) by binding to miRNAs, rendering them inaccessible for binding with mRNA. This mooching of miRNAs boosts the suppression of gene expression by miRNAs.

Following previous works, which have displayed miR-139-5p as agent of tumor suppression in CRC [15–17], lower levels of miR-139-5p have been interconnected with greater tumor size, lymph node metastases, as well as advanced tumor grade [18, 19]. Furthermore, miR-139-5p blocks Notch1 thereby hindering CRC progression [15, 20]. However, it is not well-known whether lncRNAs act together with miR-139-5p to stimulate carcinogenesis of colorectal cancer.

Here, we uncovered RP11-757G1.5, a novel lncRNA that is highly expressed in CRC tissues. Results of the Kaplan-Meier analysis, Log-rank test, Univariate as well as Multivariate analyses, all showed that up-regulated RP11-757G1.5 was linked with higher expression, infiltration and metastasis of CRC cells in vitro and in vivo. Results also showed that this was linked with poor prognosis of patients with CRC. Our outcomes demonstrated that RP11-757G1.5 acts by directly interacting with miR-139-5p, behaving as an miRNA smokescreen to epigenetically activate downstream gene YAP1 expression, and as a result this mechanism promotes proliferation and migration in patients diagnosed with CRC. As a whole, our findings illuminate the therapeutic prospect of a novel RP11-757G1.5/miR-139-5p/YAP1 axis in CRC progression in the future.

## Materials and methods

### Clinical samples

All CRC tissues and adjacent non-cancer control tissues performed in our study were collected from surgical specimens of CRC patients residing in the Department of general surgery of the second affiliated hospital of Nanchang University. All patient samples were obtained per patient’s written informed consent. Table 1 summarizes the clinical data obtained from all patients included in our study. Ethical endorsement was attained from the clinical research ethics committee of the second affiliated hospital of Nanchang University.

**Table 1:** The correlation of the expression of RP11-757G1.5 with clinical features in Colorectal cancer

Characteristics	Number of case	RP11-757G1.5	expression	***p*** value
		High(***n*** = 56)	Low(***n*** = 56)	
Gender				*p* = 0.568
Male	63 (56.3%)	30	33	
Famale	49 (43.7%)	26	23	
Age at diagnosis				*p* = 0.676
< 60	32 (28.6%)	15	17	
≥ 60	80 (71.4%)	41	39	
Differentiation				*p* = 0.404
poor	48 (42.9%)	23	25	
Moderately	28 (25.0%)	17	11	
well	36 (32.1%)	16	20	
Tumor size (cm)				***p*** = 0.003**
< 5	54 (48.2%)	19	35	
≥ 5	58 (51.8%)	37	21	
Depth of invasion				*p* = 0.693
T1,T2	40 (35.7%)	19	21	
T3,T4	72 (64.3%)	37	35	
Location				*p* = 0.779
Transverse colon	30 (26.8%)	13	17	
Ascending colon	34 (30.4%)	19	15	
Descending colon	19 (17.0%)	9	10	
Sigmoid colon	29 (25.8%)	15	14	
Lymph node status				***p*** = 0.008**
N0	52 (46.4%)	33	19	
N1 + N2	60 (53.6%)	23	37	
TNM stage				***p*** < 0.001***
I + II	61 (54.5%)	20	41	
III + IV	51 (45.5%)	36	15	

**p < 0.01; ***p < 0.001

### Reagents

Antibodies counteracting YAP1 (Rabbit mAb. Cat. No. 14074 T), Cyclin-D1 (Rabbit mAb. Cat. No. 2922S), PCNA (Rabbit mAb. Cat. No. 13110 T), E-cadherin (Rabbit mAb. Cat. No. 3195 T), N-cadherin (Mouse mAb. Cat. No. 14215S) and Ki-67 (Mouse mAb. Cat. No. 9449S) were procured from Cell Signaling Technology. Antibodies counteracting GAPDH (Rabbit mAb. Cat. No. ab8245) and Tubulin (Rabbit mAb. Cat. No. ab210797) were procured from Abcam. MiR-139-5p mimic, miR-139-5p inhibitor, pcDNA3.1-YAP1 plasmid and YAP1 siRNA (si-YAP1) were purocured from GenePharma (Suzhou, China).

### Cell culture

The following CRC cell lines were acquired from the American type culture collection (ATCC): HT-29, HCT-116, SW480, SW620, LoVo and Caco-2, and finally, the normal human colonic epithelial cell line NCM460. All cells were cultivated in DMEM (Invitrogen) and enhanced with 10% Gibco FBS (Cat. No. 10100147), 1% L-glutamine (Thermo Fisher Scientific, Cat. No. 21051024), 25 units/ml penicillin (Gibco, Cat. No. 15140148) and 25 g/ml streptomycin (Gibco, Cat. No. 15140148). All cell lines were detected and authenticated as bacteria, mycoplasma free and short tandem repeat profiling as per ATCC’s guidelines within the past 3 months. All the primers used in vector construction are shown in Table 2.

### RNA extraction and RT-qPCR assay

RNA extraction was carried out using Trizol (Invitrogen, Cat. No. 15596–026). Reverse transcription was carried out using the Superscript III transcriptase kit (Invitrogen, Cat. No. 18080–044). RT-qPCR was conducted on the Biorad CFX96 system using SYBR green. RT-qPCR was executed through the following process: 55 °C for 3 min, 95 °C for 7.5 min, and subsequently 50 cycles at 95 °C for 10 s, and 65 °C for 2 min. The next step was done at 95 °C for 2 min, 50 °C for 1 min, and 50 °C for 10 s. GAPDH was used as the reference gene. The PureLink® miRNA kit was used for mining of miRNAs. The RT-qPCR formula was as follows: 95 °C for 3 min, followed by 50 cycles at 95 °C for 10 s, and 55 °C for 50 s. The reference genes were U6 and/or β-actin. All sequences for RT-qPCR are outlined in Table 3**.**

**Table 2:** The sequences of shRNA for RP11-757G1.5

**sh-NC:** 5′-GCGGATGCCCGATTGGGCCA-3’	
**sh-RP11-757G1.5#1:** 5′-ATCCGGCGGTAGCTAGCTAAGCAA-3’	
**sh-RP11-757G1.5#2:** 5′-GAAATTGCGTACAAGGAGAGTCA-3’	
**pcDNA3.1-RP11-757G1.5**	
**F:** 5′-AGGGCCTCCGCTGGAATTTTAAAGGGTCCCTGAAG-3′ (BamHI)	
**R:** 5′-CAAGTCGAGGGTCGGGTGCAAAACAACCGATAC-3′ (XhoI)	
**miRNA-139-5p mimic:** 5′-ACCGGTTGAAAAGTGCGATGCTACCCGGTAAGGCCGCCG-3’	
**miRNA-139-5p inhibitor:**	
5′-AAGCTTGGTGGGTGGCACGCGCAAAGTGTGCAGTCATGAAGC-3’	
**si-NC:** 5′-AATTGCGGAACCGGTTGCATTAA-3’	
**si-YAP1:** 5′-GAAGGCAGTTTGAAGTGACGCGGG-3’

**Table 3:** The sequences for RT-qPCR

**GAPDH**	
**F:** 5′-TGTGGGCATCAATGGATTTGG-3’	
**R:** 5′-ACACCATGTATTCCGGGTCAAT-3’	
**RP11-757G1.5**	
**F:** 5′-CGTAGGCAAAAAGCGTACGGAT-3’	
**R:** 5′-AAAAGCGGGAAAGCGAAAGT-3’	
**miR-139-5p**	
**F:** 5′-ACACTCCAGCTGCACGTGTC-3′	
**R:** 5′-TGGTGTCGTGGAGTCGGTTGA-3’	
**YAP1**	
**F:** 5′-CGCTGACGGAGTACAAGTG-3’	
**R:** 5′-GTAGGAGCCGACCTCGTTG-3’	

### Isolation of cytoplasmic and nuclear RNA

The cytoplasmic & nuclear RNA purification kit (Norgen, Cat. No. 21000) was utilized to carry out nuclear RNA extraction and purification as per the manufacturer’s instructions.

### Plasmid construction and transfection

Sh-RP11-757G1.5#1 (sh-757G1.5#1), sh-RP11-757G1.5#2 (sh-757G1.5#2) and pcDNA3.1-RP11-757G1.5 (pcDNA3.1-757G1.5 or pcDNA3.1-) along with the respective controls (sh-NC and vector) were obtained from GenePharma (Suzhou, China). Table 2 displays shRNA sequences. 1 × 10^6^ CRC cells/well were seeded into 6-well plates and cultured until they were 50–60% confluent. These were afterwards transfected with plasmid using lipofectamine 3000 (Thermo Fisher Scientific, Cat. No. L3000008) per the manufacturer’s directions. Centrifuging at 1000 rpm for 5 min at 4 °C was utilized to gather viral particles. The particles were centrifuged and filtered then used to infect HCT-116 and SW480 cells to generate stable overexpression and knockdown cells, respectively.

### Cell proliferation assay

CRC proliferation was assessed by colony formation and Edu incorporation assays. To carry out colony formation analysis, 350 transfected cells/well were seeded into 6-well plates and cultured for 2 weeks. Next, colonies were fixed with 4% paraformaldehyde before staining with 0.5% crystal violet and the total number of colonies was counted. Edu assays were done using a commercial kit (Ribobio, Cat. No. C10310) according to the manufacturer’s instructions as described previously [21]. All assays were executed in threefold.

### In vitro cell migration and invasion assay

Cell horizontal migration was examined using wound-healing assays. When cells were approximately 95% confluent, media was extracted and a 10 μl tip used to scrape the monolayer perpendicularly. Cell fragmetns were mined from the cells by washing 3 times with PBS, after which the cells were put back in culture. The cells were imaged at 0 and 48 h after wounding using an inverted microscope (Olympus Corp, Tokyo, Japan). Wound healing capacity was determined by the size of the gaps measured under a microscope.

For cell vertical migration and invasion assays, the transfected cells were suspended once again in serum-free media and seeded into the upper chambers and then cultured for 72 h. A key point that needed extra attention was that, for cell invasion assays, 2 × 10^4^ cells were seeded in the upper chambers of transwell plates which were coated with Corning Matrigel (BD Biocoat, Cat. No. 354234) for 2 h prior to seeding, while the migration experiment was not coated with Corning Matrigel. Further Steps were the identical, 700 μl of cell culture media supplemented with 10% FCS was then added into the lower chambers and the cells incubated at 37 °C in normal cell culture conditions for 10–15 h. Cells that invaded the lower chamber were fixed with by methanol for 15 min at room temperature and stained with 0.1% (w/v) crystal violet in the dark. Assays were executed in threefold.

### Luciferase reporter assay

HCT-116 cells were co-transfected with pLuc, pRL-CMV, miR-139-5p mimic (negative control, NC), and pcDNA3.1-RP11-757G1.5 (pcDNA3.1-) and then subjected to luciferase assays using the dual-luciferase® reporter system (Beyotime, Cat. No. E1960).

### RNA pull-down assay

HCT-116 cells were lysed in 1 ml of cell lysis buffer for 72 h. 1.5 μL of RNAse inhibitor, 10 μL of streptavidin agarose beads and 500 pM of antisense oligos were added and the cells rotated overnight at 4 °C. The beads were washed 5 times using cell lysis buffer. Afterwards, purified RNAs were extracted and analyzed by RT-qPCR to illustrate the presence of binding targets.

### Ago2 immunoprecipitation assay

Transfected cells were lysed with RIPA lysis buffer and centrifuged for 20 min at 12000 rpm. 2 μl of Ago2 antibody and 10 μl of beads were added and the supernatant rotated overnight at 4 °C. The resulting mixture was rinsed 3 times with lysis buffers and RNA using Trizol reagent (Invitrogen, Cat. No. 15596–026).

### Western blotting analysis

Proteins were parted by electrophoresis on 8% or 10% SDS-PAGE gels and then transferred onto 0.45 μm PVDF membranes. The membranes were then obstructed with non-fat milk for 1–2 h at room temperature. All antibodies were diluted in Primary Antibody Dilution Buffer (Solarbio, Cat. No. A1810) at 1:1000. Finally, protein bands were perceived on X-ray film through ECL luminescence reagent (Solarbio, Cat. No. SW2010**).**

### Immunohistochemistry analysis

IHC analysis of YAP1 was executed through the Dako Envision™ FLEX + System (Dako, Glostrup, Denmark) as explained previously [21].

### In vivo studies

Thirty-two 6–8-week-old nude mice were acquired from the Shanghai laboratory animal company. HCT-116 and SW480 cells expressing a luciferase reporter (pcDNA3.1-luciferase) and stably expressing pcDNA3.1-757G1.5 and sh-757G1.5#1, were generated. Afterwards, 1 × 10^6^ HCT-116 and SW480 cells per mouse (mixed with Matrigel at a 1:1 ratio), were injected subcutaneously or intravenously for the diagnosis of tumor growth and metastasis. Tumor development and metastasis were observed weekly by means of an IVIS Fluorescent Imaging System (IVIS Spectrum). The mice were sacrificed after 6 weeks and tumors collected for analysis.

### Statistical analyses

Data are presented as the mean ± S.D. Student’s *t-*test/Unpaired two-tailed student’s *t* test, the Mann–Whitney *U*-test and the *χ2* test were utilized to analyze differences between groups. Survival rates were evaluated using Kaplan–Meier analysis and compared by the Log-rank test. HRs and 95% CIs were calculated using Cox proportional hazards model. *p*-value < 0.05 was considered statistically significant.

## Results

### Overexpression of RP11-757G1.5 in CRC associates with poor prognosis

**Fig. 1:**
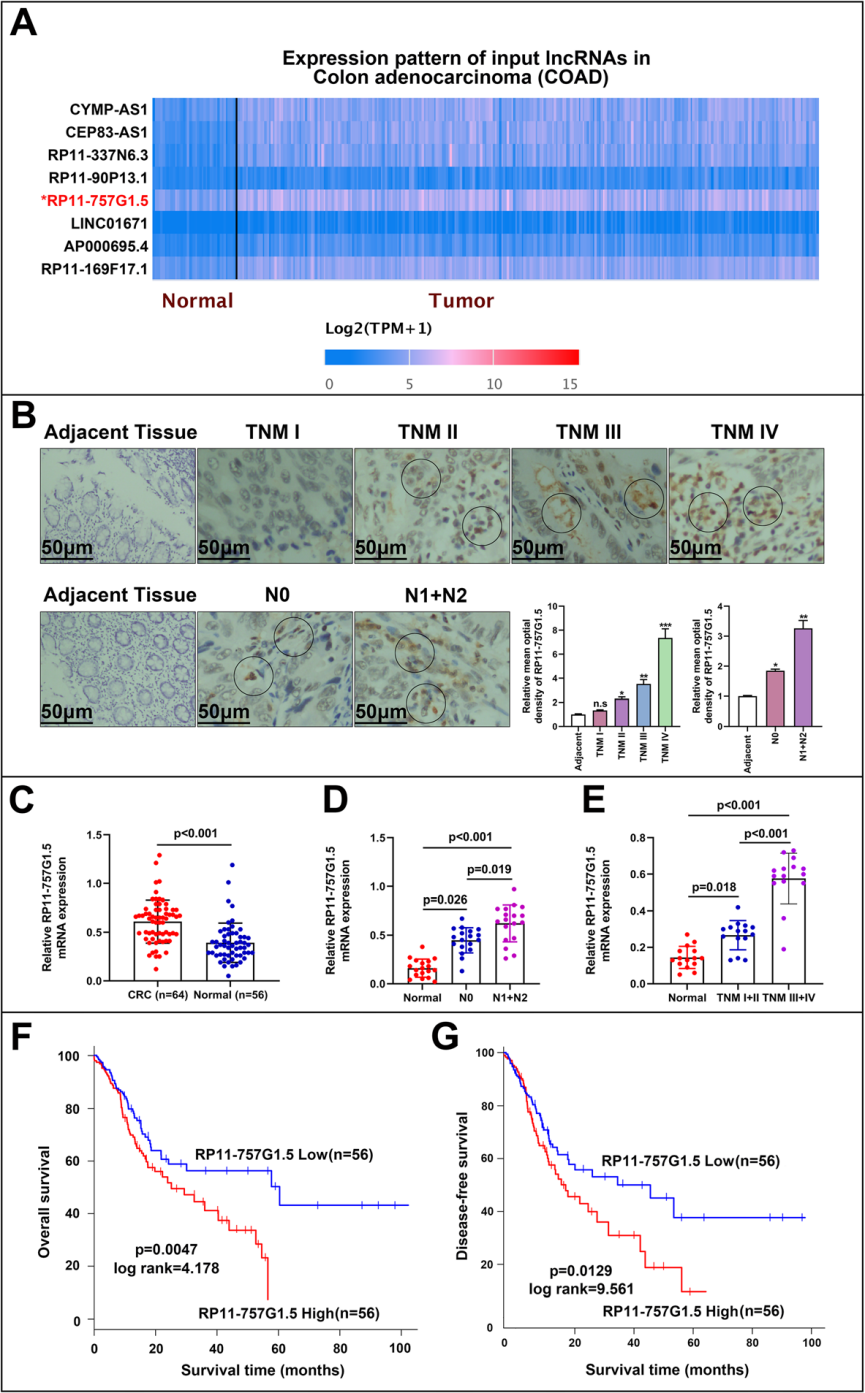
LncRNA RP11-757G1.5 expression is upregulated in CRC tissues and is associated with poor prognosis. **a** Heat-maps of lncRNAs that were differentially expressed between CRC tissues and matched adjacent normal samples. RP11-757G1.5 was the most appropriate lncRNA to select in eight lncRNAs. The color scale shown below illustrates the relative RNA expression levels; red represents high expression, and blue represents low expression. **b** The expression of RP11-757G1.5 was determined by in situ hybridization at different lymph node metastasis, TNM stages of CRC patients compare with corresponding adjacent normal tissues. Scale bar, 50 μm. **c** Comparison of RP11-757G1.5 in CRC tissues (*n* = 64) and normal tissues (*n* = 56) by RT-qPCR. **d-e** RP11-757G1.5 expression at different lymph node metastasis (Normal: normal adjacent tissues; N0: no lymph node metastasis; N1: 1 ~ 3 regional lymph node metastasis; N2: ≥4 regional lymph node metastases, *n* = 18) and TNM stages (*n* = 15) of CRC patients. **f-g** Kaplan-Meier survival analysis of the overall survival and disease-free survival in two groups defined by low and high expression of RP11-757G1.5 in patients with CRC. The median expression of RP11-757G1.5 was used as cut-off. *p* = 0.0047 and *p* = 0.0129 by Log-rank test. *p < 0.05, **p < 0.01 by Student’s *t*-test. Data are representative of at least three independent experiments

To further ascertain lncRNAs that are differentially expressed in CRC, we primarily analyzed GSE63675 from GEO, which comprises of lncRNA data for 43 CRC tissues and 6 neighboring non-tumor control tissues. Of note, CRC tissues expressed 8 notably differentially expressed lncRNAs as compared with neighboring non-tumor tissues (Fig. 1a). Amid them, lncRNA RP11-757G1.5 was chosen for further analysis. Principally, in situ hybridization (ISH) was applied to gauge level of the expression of RP11-757G1.5 in tissues. As presented in Fig. 1b, RP11-757G1.5 was gradually strongly stained with staging and lymph node metastasis in CRC tissues comparing to adjacent tissues. Also, RP11-757G1.5 was intensively stained in CRC cells’ cytoplasm. Next, we assessed the expression of RP11-757G1.5 in CRC tissues by RT-qPCR and it was ascertained that this lncRNA was markedly upregulated in CRC tissues relative to the non-tumor control tissue (*p* < 0.001, Fig. 1c). Successively, we appraised the link between high RP11-757G1.5 expression and clinicopathological features of the disease. Results were similar to ISH and pointed out that elevated RP11-757G1.5 expression was directly linked with significant lymph node metastasis and advanced TNM staging (Fig. 1d, e, S1A and S1B). To assess the importance of this relationship, we distributed 112 CRC patients into 2 sets in relation to the extent of RP11-757G1.5 expression: RP11-757G1.5-high and RP11-757G1.5-low. Pearson chi-square or Fisher’s Exact tests revealed that elevated RP11-757G1.5 levels were linked with greater tumor size (*p* = 0.003), lymph node metastasis (*p* = 0.008) and advanced TNM staging (*p* < 0.001). A relationship between RP11-757G1.5 levels and other clinical features was not observed (Table 1). Kaplan-Meier analyses and Log-rank tests were subsequently carried out to establish the connection between RP11-757G1.5 and CRC survival time. Resultls of this evaluation showed that patients in the RP11-757G1.5-high group displayed a remarkably shorter survival rate relative to those in the RP11-757G1.5-low group (45.741 ± 3.539 vs 69.818 ± 3.662 months; Log rank = 4.178, *p* = 0.0047, Fig. 1f). Moreover, elevated RP11-757G1.5 levels were positively associated with poor disease-free survival (Log rank = 9.561, *p* = 0.0129, Fig. 1g). Univariate and multivariate analyses revealed RP11-757G1.5 expression as an autonomous prognostic sign of CRC (hazard ratio (HR) =3.441, 95% confidence interval (CI) =1.471–8.005, *p* = 0.008; HR = 2.015, 95% CI = 1.018–5.856, *p* = 0.019, Table 4). Wholly, these findings recognized that high RP11-757G1.5 levels correlate with poor CRC clinical outcomes.

**Table 4:** Multivariate analysis of clinicopathological factors for disease-specific survival

Variable	Subset	Univariateanalysis		Multivariate analysis	
		***p***-value	HR (95% CI)	***p***-value	HR (95% CI)
**Gender**	**Male/female**	**0.771**	**0.734 (0.415–1.859)**	**–**	**–**
**Age at diagnosis (years)**	**< 60/≥60**	**0.474**	**0.626 (0.537–2.552)**	**–**	**–**
**Differentiation**	**Well + moderately/poorly**	**0.331**	**1.422 (0.614–2.683)**	**–**	**–**
**Tumor size (cm)**	**< 5/≥5**	0.002**	**2.742 (0.326–4.663)**	0.017*	**3.471 (0.527–6.028)**
**Depth of invasion**	**T1 + T2/T3 + T4**	**0.095**	**1.116 (0.549–4.241)**	**–**	**–**
**Location**	**Colon/rectum**	**0.852**	**0.632 (0.412–1.743)**	**–**	**–**
**Lymph node status**	**N0/N1 + N2**	**0.153**	**2.571 (1.306–4.663)**	**0.216**	**2.154 (1.146–6.915)**
**TNM stage**	**I + II/III + IV**	0.003**	**13.145 (5.044–34.378)**	0.004**	**7.603 (2.521–33.109)**
**RP11-757G.15**	**High/low**	0.008**	**3.441 (1.471–8.005)**	0.019*	**2.015 (1.018–5.856)**

* *p* < 0.05; **p < 0.01; ***p < 0.001

### RP11-757G1.5 promotes CRC cell proliferation and cell cycle progression in vitro

**Fig. 2:**
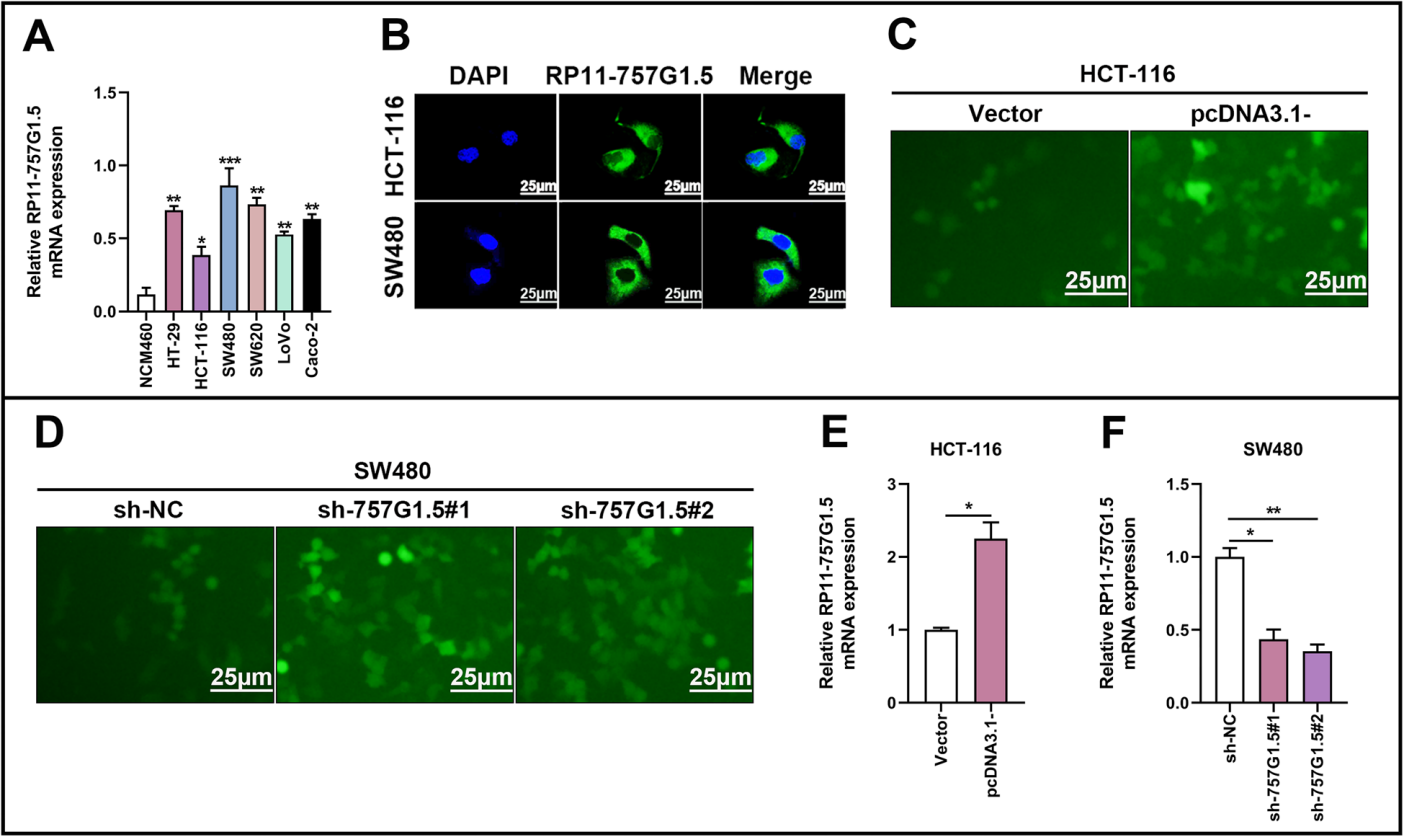
Overexpression and stable knockdown of RP11-757G1.5 in HCT-116 and SW480 cells. **a** Relative expression of RP11-757G1.5 in six CRC cell lines and a colonic epithelial cell. **b** Fluorescence in situ hybridization (FISH) assay was conducted to determine the subcellular localization of RP11-757G1.5 in HCT-116 and SW480 cells. Nuclei are stained blue (DAPI), and RP11-757G1.5 are stained green. Scale bar, 25 μm. **c-d** Representative images of HCT-116 and SW480 cells transfected with pcDNA3.1-757G1.5 and sh-757G1.5#1; sh-757G1.5#2, respectively. Scale bar, 25 μm. **e-f** The validation of overexpression and knockdown efficacy of RP11-757G1.5 in CRC cell lines by RT-qPCR. *p < 0.05, **p < 0.01 by Student’s *t*-test, Data are representative of at least three independent experiments

**Fig. 3:**
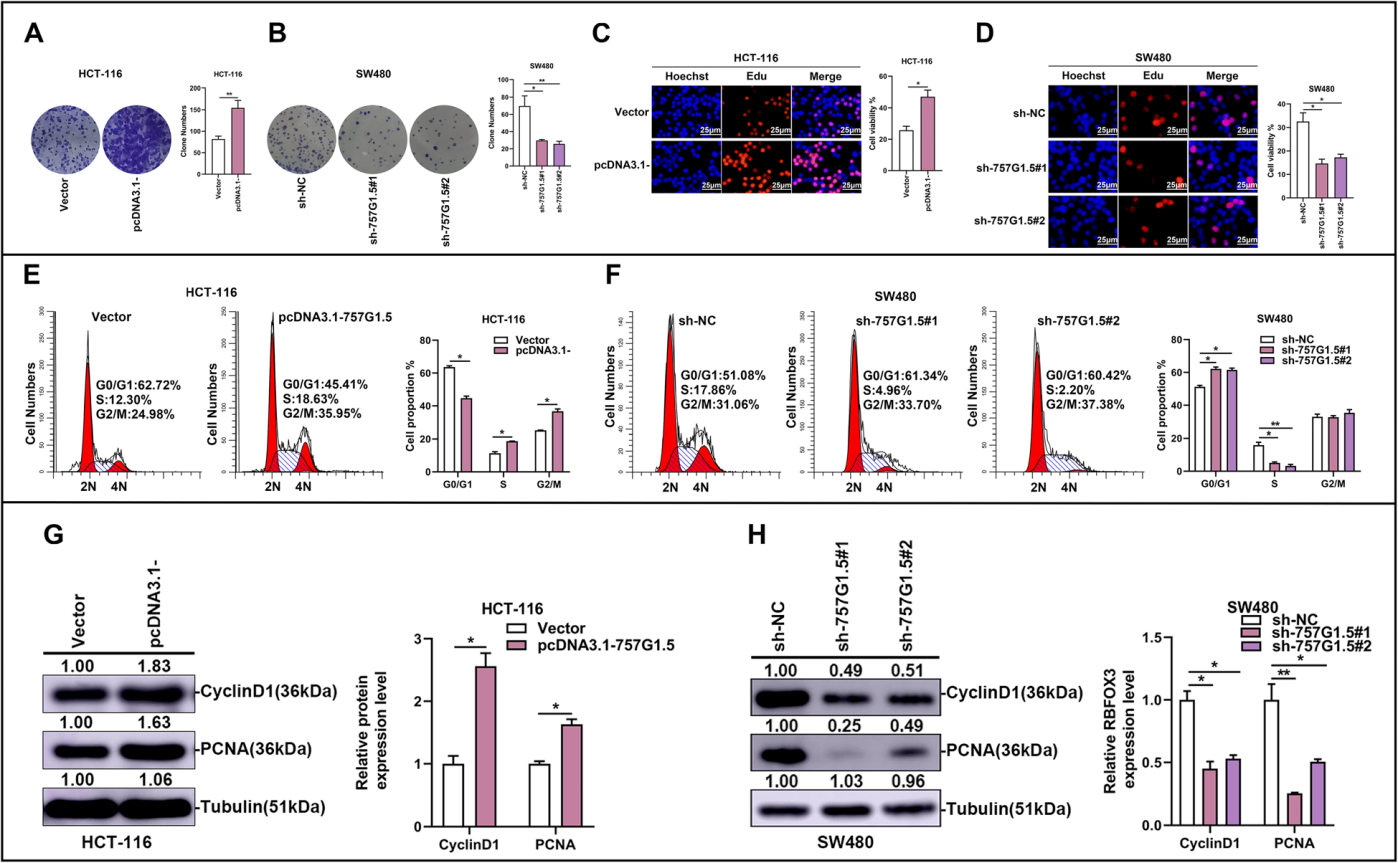
RP11-757G1.5 promotes CRC cell proliferation in vitro. **a-b** Effects of RP11-757G1.5 overexpression and knockdown on colony formation in CRC cells. **c-d** Effects of RP11-757G1.5 overexpression and downregulation on CRC cell proliferation were measured by a Edu assay. Scale bar, 25 μm. **e-f** Cell cycle analysis was conducted in HCT-116 and SW480 cells transfected with pcDNA3.1-757G1.5 and sh-757G1.5#1; sh-757G1.5#2, respectively. **g-h** Western blotting was performed to detect proliferation-associated antigen Cyclin D1 and PCNA expression in HCT-116, SW480 cells transfected with pcDNA3.1-757G1.5 or sh-757G1.5, respectively. *p < 0.05, **p < 0.01 by Student’s *t*-test. Data are representative of at least three independent experiments

Next, we assessed the expression pattern of RP11-757G1.5 in standard NCM460 and CRC cell lines (HT-29, HCT-116, SW480, SW620, LoVo, Caco-2) by RT-qPCR. The outcomes revealed markedly higher levels of RP11-757G1.5 in CRC cell lines compared to NCM460 (*p* < 0.05, Fig. 2a). RP11-757G1.5 was expressed was most in SW480 (*p* < 0.001) and least in HCT-116 (*p* < 0.05). These 2 CRC cell lines were therefore selected for downstream experiments. In accordance, the results of fluorescence in situ hybridization (FISH) also pointed out that lncRNA RP11-757G1.5 is mainly found in the cytoplasm (Fig. 2b). The possible biological function of RP11-757G1.5 in CRC was evaluated by observing the overexpression of RP11-757G1.5 in HCT-116 cells (Fig. 2c), while it was knocked down in SW480 cells (Fig. 2d). RT-qPCR was conducted to confirm the success of the evaluation, and Fig. 2e and f (*p* < 0.05) show the outcomes. To lessen the likelihood of off-targeting, 3 shRNAs (sh-RP11-757G1.5#1 (or sh-757G1.5#1) and sh-RP11-757G1.5#2 (or sh-757G1.5#2)) were used (Fig. 2d). Further analyses showed that RP11-757G1.5 overexpression significantly enhanced HCT-116 colony formation and proliferation while these processes were suppressed by RP11-757G1.5 silencing in SW480 cells (*p* < 0.05, Fig. 3a-d). In addition, flow cytometry showed that RP11-757G1.5 silencing significantly hindered the SW480 cell cycle at the G1 phase (*p* < 0.05, Fig. 3f). RP11-757G1.5 overexpression in HCT-116 cells had similar results (*p* < 0.05, Fig. 3e). Furthermore, immunoblotting analysis demonstrated that overexpression of RP11-757G1.5 in HCT-116 enhanced Cyclin D1 and PCNA expression, factors known to stimulate cell proliferation (*p* < 0.05, Fig. 3g), while RP11-757G1.5 knockdown weakened their expression (*p* < 0.05, Fig. 3h). In other words, RP11-757G1.5 promotes cell proliferation of CRC by regulating Cyclin D1 and PCNA signaling pathway. However, its specific molecular mechanism needs further study. As a whole, these datasets concluded that RP11-757G1.5 might serve as a cancer-promoting factor, which stimulates the proliferation of CRC cells in vitro.

### RP11-757G1.5 promotes CRC cell migration and invasion in vitro

**Fig. 4:**
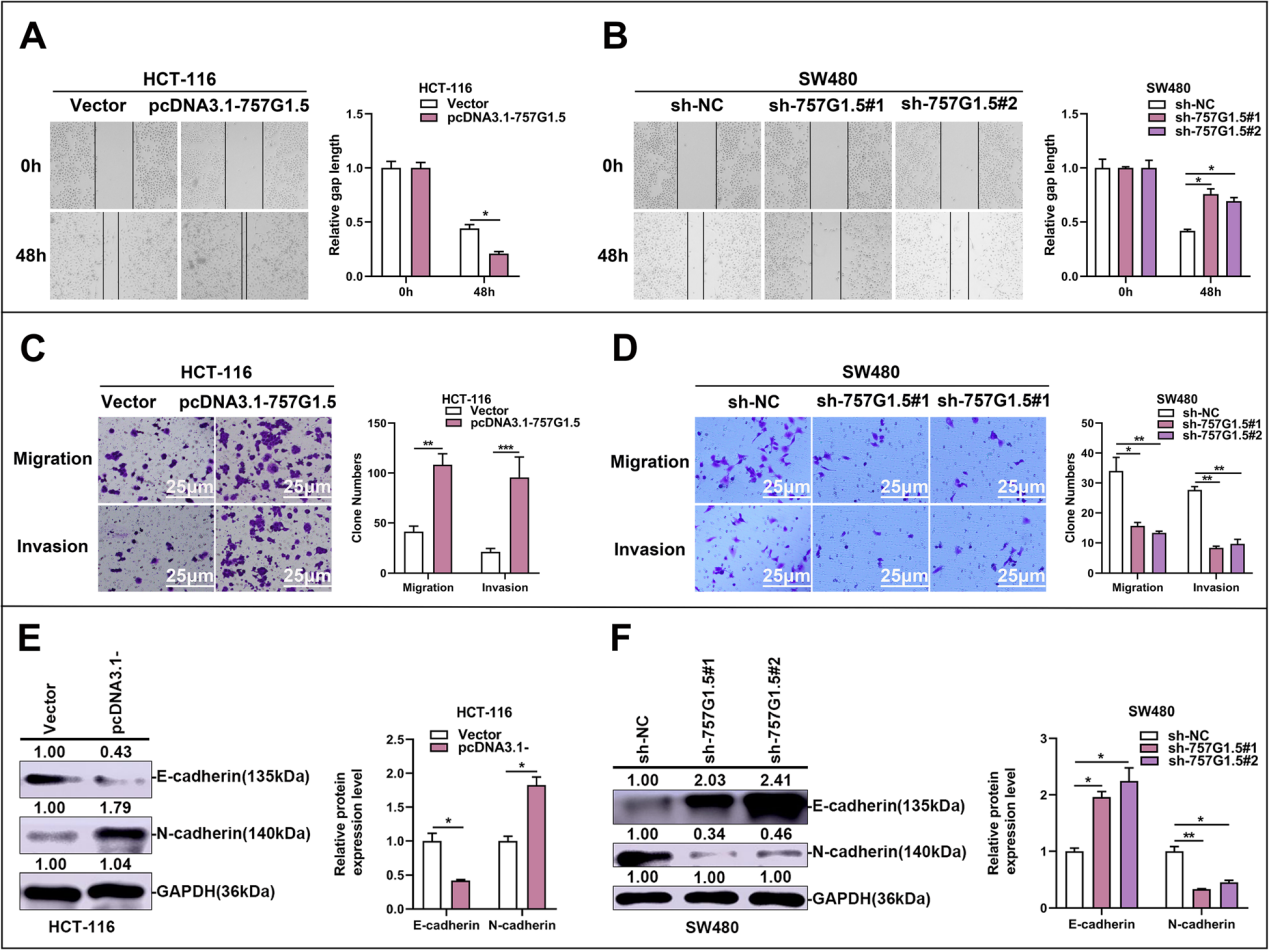
RP11-757G1.5 accelerates CRC cell migration and invasion in vitro**. a-b** Wound-healing assay was used to investigate the horizontal migration ability with RP11-757G1.5 overexpression or knockdown in CRC cells, and relative gap distance was calculated and plotted on a histogram. **c-d** The transwell assays were applied in both HCT-116 and SW480 cells to evaluate the CRC cell vertical migration and invasion abilities prior transfected with pcDNA3.1-757G1.5 or sh-757G1.5, respectively, and the number of cells was calculated and plotted on a histogram. Scale bar, 25 μm. **e-f** Immunoblotting analysis was performed to determine epithelial-mesenchymal transition marker E-cadherin and N-cadherin expression in HCT-116, SW480 cells transfected with pcDNA3.1-757G1.5 or sh-757G1.5, respectively. *p < 0.05, **p < 0.01, ***p < 0.001 by Student’s *t*-test. Data are representative of at least three independent experiments

The role of RP11-757G1.5 in CRC metastasis was determined by cell invasion and migration assays. The results show that RP11-757G1.5 overexpression facilitated HCT-116 cell migration and incursion (*p* < 0.05, Fig. 4a, c). On the contrary, RP11-757G1.5 knockdown suppressed these processes in SW480 cells (*p* < 0.05, Fig. 4b, d). So, it is suggested that RP11-757G1.5 is involved in cancer cells dissemination in vitro. Further, we executed western blot to estimate whether RP11-757G1.5 controls epithelial-mesenchymal transition (EMT) in CRC cells. The outcomes illustrated that RP11-757G1.5-overexpression leads to a reduction in the expression of epithelial cell marker E-cadherin and an augmention in the expression of another epithelial cell marker N-cadherin (*p* < 0.05, Fig. 4e, f). Therefore, we initially verified RP11-757G1.5 promotes metastasis through affecting the EMT process of CRC cells. Moreover, to further verify the cancer-promoting effect of RP11-757G1.5 in CRC. We selected SW620 (High metastasis) and HT-29 (Low metastasis) two cell lines of CRC, then successfully performed RP11-757G1.5 knockdown and overexpression, respectively. The results confirmed that RP11-757G1.5 acts as an oncogene which could promote metastasis of CRC (*p* < 0.05, Figure S4A, S4B). Collectively, these results strongly propose that the RP11-757G1.5 can prompt migration and invasion of CRC cells in vitro*.*

### LncRNA RP11-757G1.5 acts as a molecular sponge of miR-139-5p to regulate YAP1 expression in CRC cells

**Fig. 5:**
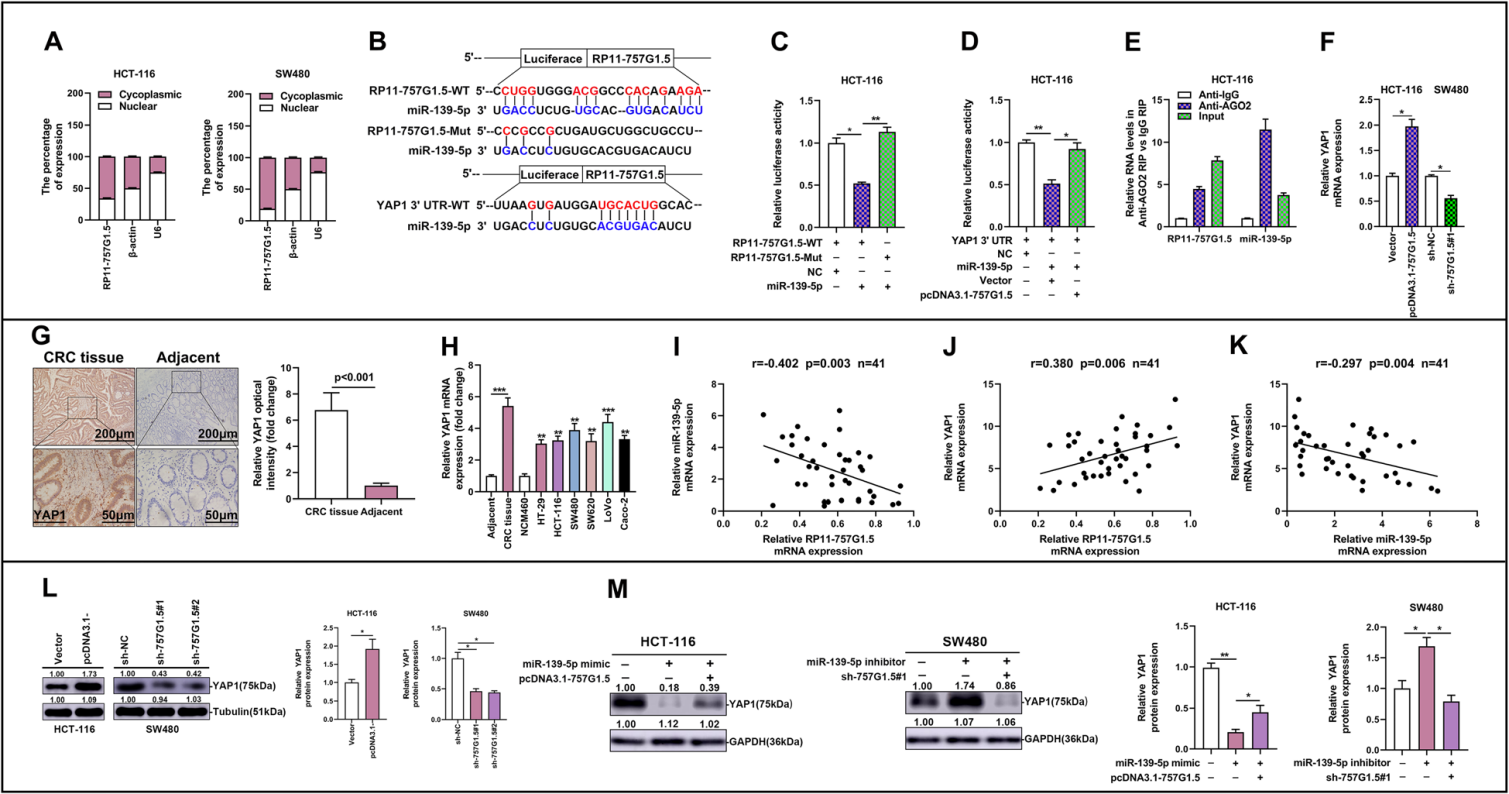
RP11-757G1.5 sponges miR-139-5p and modulate YAP1 expression. **a** Subcellular localization of RP11-757G1.5 was determined by RT-qPCR in HCT-116 and SW480 cell lines. **b** miR-139-5p-binding sequence in RP11-757G1.5 and YAP1 3’UTR. A mutation was generated in RP11-757G1.5 in the complementary site for miR-139-5p binding. **c** Luciferase activity of a luciferase reporter plasmid (pLuc) containing wild-type or mutant RP11-757G1.5 co-transfected with miR-139-5p was determined using the dual luciferase assay. **d** MiR-139-5p and pLuc plasmid containing YAP1 3’UTRs were co-transfected with pcDNA3.1-757G1.5 or empty vector into HCT-116 cells to verify whether RP11-757G1.5 can function as a ceRNA of miR-139-5p. **e** Cellular lysates from HCT-116 cells were used for RIP with an anti-Ago2 antibody or IgG antibody. The levels of RP11-757G1.5 and miR-139-5p were detected by RT-qPCR. **f** and **l** The expression levels of YAP1 in HCT-116 cells transfected with pcDNA3.1-757G1.5 and SW480 cells transfected with sh-757G1.5 were analyzed by RT-qPCR and western blotting. **g** The elevated expression of YAP1 in tissue level was detected by immunohistochemistry (IHC) test, normalized to para-tumor tissue group. Scale bar, 200 μm; 50 μm. **h** YAP1 was up-regulated in CRC tissue and cell lines as determined by a RT-qPCR, normalized to para-tumor tissue group and NCM460 group, respectively. **i-k** Correlation between RP11-757G1.5, miR-139-5p, and YAP1 expression in CRC and normal colon specimens as detected by RT-qPCR (*n* = 41). **m** Western blot assays were performed to test YAP1 expression after HCT-116 cells were transfected with miR-139-5p mimic or co-transfected with miR-139-5p mimic and pcDNA3.1-757G1.5. Meanwhile, SW480 cells were transfected with miR-139-5p inhibitor or co-transfected with miR-139-5p inhibitor and sh-757G1.5#1. Data from western blot assay has been represented as a quantification graph normalized to the levels of GAPDH together with the statistical tests. *p < 0.05, **p < 0.01, ***p < 0.001 by Student’s *t*-test. Data are representative of at least three independent experiments

To comprehend the mechanisms by which RP11-757G1.5 fuels CRC growth, we appraised its subcellular locality in HCT-116 and SW480 and perceived that it principally resided in the cytosol (Fig. 5a). From this we can deduce that it may function at the post-transcriptional level. Various studies have informed that lncRNAs may act as a sponge by binding competitively to miRNA, and in this manner modulating gene expression by making it impossible for target miRNA responsive elements (MREs) to bind with the miRNAs. To determine whether RP11-757G1.5 also plays this role, we cloned the full-length RP11-757G1.5 comprising the presumptive miR-139-5p binding sites in order to examine potential binding sites between miR-139-5p and RP11-757G1.5 (Fig. 5b). This analysis was done with the knowledge that miR-139-5p and RP11-757G1.5, express opposing functions in CRC. We therefore created a luciferase reporter plasmid (pLuc) containing RP11-757G1.5 (pLuc-RP11-757G1.5-WT) or a mutant bearing mutation in the miR-139-5p seed sequence (pLuc-RP11-757G1.5-Mut). Through luciferase reporter assays, we observed the link bewteen miR-139-5p and RP11-757G1.5. Results showed that overexpressing miR-139-5p (or its miR-139-5p mimic) markedly suppressed pLuc-RP11-757G1.5-WT activity relative to the mutant reporter (*p* < 0.05, Fig. 5c). This confirmed that miR-139-5p binds and inhibits RP11-757G1.5. To investigate whether this interaction was physical, we carried out RNA immunoprecipitation (RIP) with an anti-Ago2 antibody. As a result, we found enrichment for both RP11-757G1.5 and miR-139-5p in the Ago2 complex (Fig. 5e). In the meantime, miR-139-5p was pulled down by biotin-labeled RP11-757G1.5-WT (*p* < 0.01, Figure S5B), while mutagenesis of the binding sites for miR-139-5p in RP11-757G1.5-Mut disrupted the interaction between RP11-757G1.5 and miR-139-5p (*n.s*, Figure S5B).

### RP11-757G1.5 modulates YAP1 expression by competitively binding miR-139-5p

Previous investigations have proved that microRNAs control CRC pathogenesis via YAP1 regulation [22–24]. By means of bioinformatics analysis, miRNAs sequences were found to be compatible with recognition sequences on RP11-757G1.5 and the 3′-UTR of YAP1. Similarly, miR-139-5p, miR-4438, miR-92a-3p, miR-135b-3p and miR-203a-3p are displayed potential miRNAs targets as well as binding sites of RP11-757G1.5 from bioinformatics analysis (Figure S5A). Then, RT-qPCR experiment was preformed to identify the expression profile of the abovementioned miRNAs in CRC-HCT116 cells after transfected by pcDNA-RP11-757G1.5. The results demonstrated significantly decreased miR-139-5p expression following overexpressing of RP11-757G1.5 (*p* < 0.05). However, the expression levels of the other four miRNAs did not altered dramatically. (Figure S5A). This suggested that miR-139-5p posssibly explicitly interacted with the RP11-757G1.5 sequence and its 3′-UTR (Fig. 5b). Following this, we attempted to elucidate whether the effects of RP11-757G1.5 on CRC pathogenesis are facilitated by the miR-139-5p/YAP1 pathway. Post evaluating the relationships between RP11-757G1.5, miR-139-5p, and YAP1 by luciferase assays, it was seen that, in comparison to the empty vector control, RP11-757G1.5 overexpression reversed the miR-139-5p-mediated inhibition of pLuc-NOTCH1–3’UTR luciferase output (*p* < 0.05, Fig. 5d), thus indicating that RP11-757G1.5 suppresses miR-139-5p-mediated inhibition of YAP1 by competitively binding miR-139-5p. An elevation of YAP1 in CRC tissues and cell lines was further observed (*p* < 0.01, Fig. 5g, h). RP11-757G1.5 knockdown significantly suppressed endogenous YAP1 levels in CRC cells (*p* < 0.05, Fig. 5f, l). Counter wise, YAP1 expression was raised by RP11-757G1.5 overexpression in CRC cells (*p* < 0.05, Fig. 5f, l). We then analyzed our RT-qPCR results to gauge the relationship between RP11-757G1.5, miR-139-5p, and YAP1 expression. The results indicated that RP11-757G1.5 expression negatively correlated with miR-139-5p levels (*r* = − 0.402, *p* = 0.003), but positively correlated with YAP1 levels (*r* = 0.380, *p* = 0.006). Furthermore, a negative relationship was discovered between miR-139-5p and YAP1 expression in our RT-qPCR dataset (*r* = − 0.297, *p* = 0.004) (Fig. 5i-k). We investigated the possibility of the effects of RP11-757G1.5 on YAP1 levels depended on miR-139-5p by co-transfecting HCT-116 cells with the miR-139-5p mimic and pcDNA3.1-RP11-757G1.5 (or pcDNA3.1-757G1.5) and the effects of this approach on YAP1 were assessed. Results exhibited significantly raised protein levels of YAP1 in HCT-116 cells in relation to cells transfected with miR-139-5p mimic. RP11-757G1.5 knockdown distinctly reversed the inhibitory effects of miR-139-5p on YAP1 expression in SW480 cells, as revealed by western blot analysis (*p* < 0.05, Fig. 5m).

### RP11-757G1.5 promotes tumor progression in CRC via miR-139-5p/YAP1 axis

**Fig. 6:**
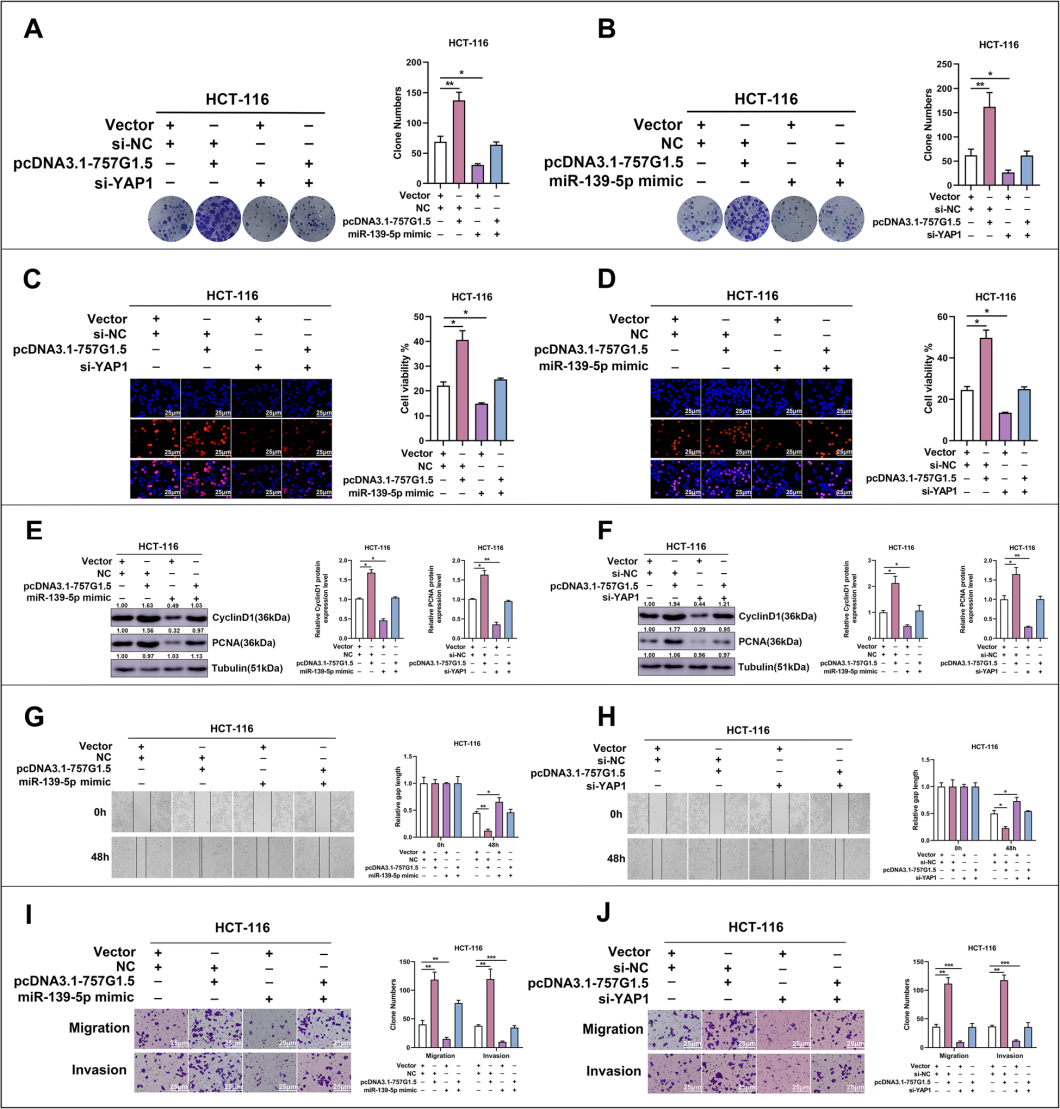
RP11-757G1.5 exerts tumor-promoting function in CRC by regulating the miR-139-5p/YAP1 axis. **a-d** The increased cell proliferation ability in pcDNA3.1-757G1.5 transfected CRC cells was abolished by ectopic miR-139-5p expression or YAP1 knockdown. The cell proliferation ability was measured by a colony formation and Edu proliferation assay. Scale bar, 25 μm. **e** Western blotting analysis of proliferation-associated antigens in HCT116 cells transfected with miR-139-5p mimic or co-transfected with miR-139-5p mimic and pcDNA3.1-757G1.5. **f** Western blotting was used to determine the changes of the proliferation-associated antigens in HCT-116 cells transfected with si-YAP1 (siRNA) or co-transfected with si-YAP1 and pcDNA3.1-757G1.5. Tubulin served as a loading control. **g-j** The increased cell migration and invasion ability in pcDNA3.1-757G1.5 transfected CRC cells were abolished by ectopic miR-139-5p expression or YAP1 knockdown. Cell migration and invasion were measured by wound healing and transwell assays. Scale bar, 25 μm. *p < 0.05, **p < 0.01, ***p < 0.001 by Student’s *t*-test. The data represented from at least three independent experiments

Based primarily on the above experiment, we provided strong evidence which indicated RP11-757G1.5 as a key controller of the expression of YAP1 by sponging miR-139-5p in CRC. In formerly published studies, miR-139-5p and YAP1 have been described to regulate CRC development [15, 25–27]. Therefore we speculated the possibility of RP11-757G1.5 regulating the pathogenesis of CRC via the miR-139-5p/YAP1 axis. To examine this, we assessed how miR-139-5p and YAP1 affect RP11-757G1.5-driven cell proliferation. Results of our investigation demonstrated that miR-139-5p overexpression or YAP1 knockdown obstructed RP11-757G1.5-driven CRC cell proliferation (*p* < 0.05, Fig. 6a-d). Subsequently, western blotting was performed in order to evaluate whether miR-139-5p and YAP1 have an effect on Cyclin D1 and PCNA levels in the context of RP11-757G1.5-driven cell proliferation. Results revealed the effects of RP11-757G1.5 overexpression on Cyclin D1 and PCNA expression were markedly reversed by miR-139-5p expression or YAP1 knockdown (*p* < 0.05, Fig. 6e, f). Following this, we assessed how the miR-139-5p/YAP1 axis affected RP11-757G1.5 driven migration and infiltration of CRC cells. The outcome showed that miR-139-5p expression or YAP1 silencing attenuated cell migration and invasion as a result of RP11-757G1.5 overexpression in CRC cells (*p* < 0.05, Fig. 6g-j). Meanwhile, we evaluated whether YAP1 can affect miR-139-5p-driven cell proliferation and metastasis in CRC. This analysis exposed the overexpression of YAP1 as the cause of the restoration of miR-139-5p-driven CRC cell proliferation and metastasis (*p* < 0.05, Figure S6A-H). This was consistent with previous studies. Comprehensively, data demonstrated that RP11-757G1.5 behaves as an oncogene in CRC to promote proliferation and metastasis, partly by sponging miR-139-5p, thereby regulating YAP1 levels.

### Deregulation of lncRNA RP11-757G1.5 suppresses cell proliferation and invasion in CRC orthotopic xenografts

**Fig. 7:**
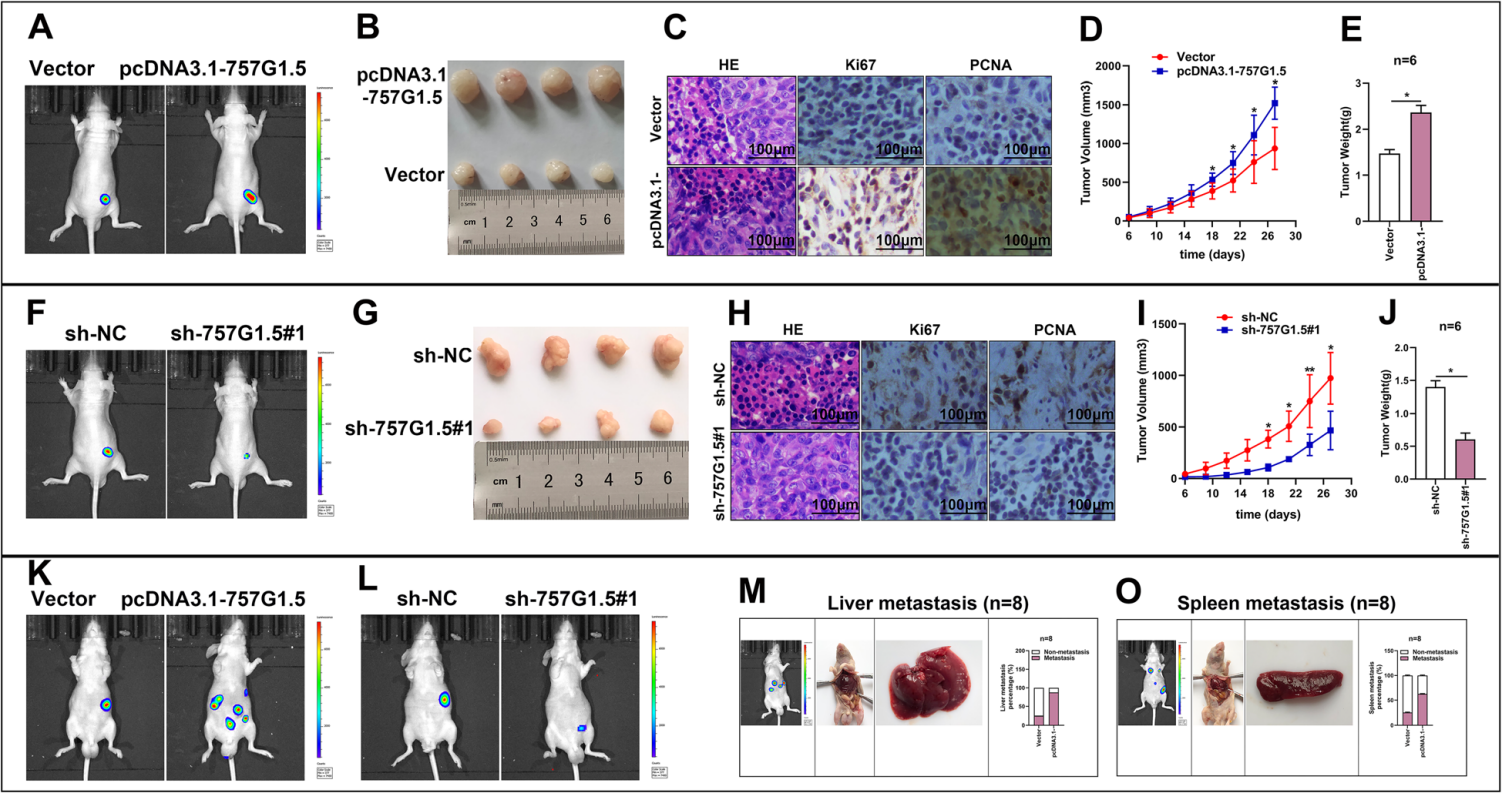
Deregulation of lncRNA RP11-757G1.5 suppresses CRC cell proliferation and invasion in CRC orthotopic xenografts. **a-e** Representative IVIS images of tumor size (**a**), macroscopic appearance (**b**), tumor growth curves (**c**), tumor weight (**d**), and metastasis (**e**) in pcDNA3.1-757G1.5 group vs control group. Scale bar, 100 μm. **f-j** Representative IVIS images of tumor size (**f**), macroscopic appearance (**g**), tumor growth curves (**h**), tumor weight (**i**), and metastasis (**j**) in sh-757G1.5#1 group vs control group. **k-l** Representative macroscopic appearance and IVIS image of metastatic foci (white arrows) in the liver (**k**) and spleen (**l**). Each sample was run in triplicate and in multiple experiments. Data are presented as mean ± SEM. *p < 0.05, **p < 0.01 by Student’s *t*-test. Data were shown as mean ± SD for three independent experiments

In this section, we explored the role of RP11-757G1.5 in CRC in vivo by building orthotopic xenograft mouse models. In short, HCT-116 cells overexpressing RP11-757G1.5 or with SW480 knockdown were xenografted subcutaneously into mice. The stimulating effect of RP11-757G1.5 knockdown on tumor evolution and migration was inspected by use of the IVIS imaging system. Results of this investigation showed that tumor luciferase activity in the pcDNA3.1-757G1.5 expressing cells was elevated in those transfected with an empty vector (Fig. 7a). Meanwhile, RP11-757G1.5-silencing exhibited approximately opposite effects (Fig. 7f). Moreover, we discovered that RP11-757G1.5 overexpression enhanced tumor growth (Fig. 7a-e). RP11-757G1.5 knockdown hindered tumor growth (Fig. 7f-j). In addition, overexpression of RP11-757G1.5 stimulated metastasis to the liver and spleen. However, when interfering with RP11-757G1.5, the metastasis of tumor cells to organs in the liver and spleen was significantly reduced (Fig. 7k-o). Evaluating the results, we could see that the elevation of RP11-757G1.5 behaves as an oncogene and contributes to tumorigenesis and liver, spleen metastasis of CRC in vivo.

## Discussion

Accumulating evidences have greatly underscored lncRNAs are tumor-associated biological molecules and have been found to serve multiple functions in the tumorigenesis and progression of CRC. Apart from well characterized lncRNAs, HOTAIR [28], MALAT1 [29] and H19 [30], the investigation of other prospective essential lncRNAs that play a role in CRC pathogenesis is also worthwhile. To ascertain these potential functional lncRNAs, we initially considered openly available CRC-associated microarray data. A novel lncRNA RP11-757G1.5 was discovered, which was seen to be greatly expressed both in CRC tissues and cell lines relative to the findings in adjacent non-cancer tissues. Followed by, the associations of RP11-757G1.5 with clinic and pathological characteristics were analyzed. Moreover, their effects on prognosis were analyzed by the Kaplan-Meier analysis, Log-rank test, Univariate and Multivariate Cox regression analysis. Meaningfully, our data implies that larger tumor size is positively correlated with the higher expression of RP11-757G1.5, along with lymph node metastases, high TNM staging, and poor clinical outcomes of CRC. In tumor evolution, lncRNAs regulate a wide array of biological processes, including tumor growth and progression of CRC. As such, this RNA group has the likelihood to be beneficial for application in the diagnosis, treatment, and prediction of CRC clinical outcomes. Subsequently, to further distinguish the effect of RP11-757 g1.5 on the biological behavior of CRC cells, we carried out cell proliferation and cell metastasis experiments, including Colony formation, Edu cell proliferation, Flow cytometry, Wound healing and Transwell assays. Loss-of-function assays displayed that subdual of RP11-757G1.5 markedly inhibited CRC proliferation, invasion, and migration in vitro*.* On the contrary, RP11-757G1.5-overexpressing promoted these processes. The mechanism is related to the regulation of Cyclin D1 and PCNA to promote proliferation, E-cadherin and N-cadherin to enhance cell metastasis. Nevertheless, more specific molecular mechanism needs further study. In vivo experiments establish that deregulation of RP11-757G1.5 represses cell accretion and infiltration in CRC orthotopic xenografts. Furthermore, overexpression of RP11-757G1.5 stimulated metastasis to the liver and spleen. Overall, these results suggested RP11-757G1.5 as an oncogene in CRC.

LncRNAs have been advocated to exercise their biological tasks by behaving as ceRNAs, which by sponging miRNAs render them inaccessible for interaction with target miRNA. For instance, the lncRNA DANCR, has been described to behave as a ceRNA for miR-335-5p and miR-1972, thereby enhancing ROCK1-mediated osteosarcoma pathogenesis [31]. In hepatocellular carcinoma, the lncRNA MCM3AP-AS1 could enhance cancer development by affecting the miR-194-5p/FOXA1 axis [32]. In other studies, LINC00152 was described to promote CRC proliferation, metastasis, and 5-Fu resistance by impeding miR-139-5p [15]. A different study found that the substantial downregulation of miR-139-5p in CRC tissues inhibited CRC growth, proliferation and metastasis while in contrast encouraged cell death and cell cycle arrest by targeting Notch1 signaling [20]. There are an array of microRNAs which have been stated to encourage CRC carcinogenesis by regulating YAP1 signaling [24, 33, 34]. Consequently, in the present study we hypothesized that lncRNA RP11-757G1.5 may regulate CRC advancement by targeting miR-139-5p-YAP1 action. To assess the likelihood of this, we used bioinformatics analysis and luciferase reporter assays to assess the interaction between possible MREs and RP11-757G1.5. Predictably, we saw that miR-139-5p did repress the binding of RP11-757G1.5-WT to its targets, while not RP11-757G1.5-Mut. Investigation of the subcellular locality of RP11-757G1.5 in CRC cells showed that it was in a position in the cytosol similar to that of miR-139-5p. In addition, RP11-757G1.5 silencing distinctly enhanced miR-139-5p levels in CRC cells. Additional study revealed that RP11-757G1.5 levels inversely associated with miR-139-5p levels. Subsequent luciferase reporter assays, Ago2 immunoprecipitation assays and RNA pull-down showed that RP11-757G1.5-WT physically interacted with miR-139-5p, thereby sponging miR-139-5p in molecular level. Moreover, we have presented convincing data to support that RP11-757G1.5 can encourage the proliferation and metastasis of CRC cells by sponging miR-139-5p in vitro.

YAP1 plays a part in several cellular processes and cancers [35–37]. However, it is still unclear by which mechanism lncRNA regulates the function of YAP1. Here, we identified YAP1 as a direct target of miR-139-5p in CRC by luciferase reporter assays. Particularly, we found that RP11-757G1.5 and miR-139-5p display contrasting functions in CRC, with RP11-757G1.5 stimulating cancer progression and miR-139-5p subduing it. In CRC, a positive correlation was also observed between RP11-757G1.5 and YAP1, while an inverse link was found between miR-139-5p and YAP1. Restoration of YAP1 suppressed the proliferation, migration and invasion of cells induced by RP11-757G1.5 knockdown. Finally, in an in vivo animal study, we proved once more that RP11-757G1.5 positively triggered tumor growth and liver, spleen metastasis in mice. Unfortunately, it is uncertain whether the RP11-757G1.5/miR-139-5p/YAP1 pathway is involved in the CRC tumorigenesis in vivo.

The pathogenesis of CRC is a multi-step and slow biological process which involves a vast number of molecular events and complicated mechanisms. The ceRNA network which is comprised by RP11-757G1.5/miR-139-5p/YAP1 is just the tip of an iceberg in colorectal cancer. We have cause to consider that multitudinous downstream targets and more precise mechanisms of RP11-lncRNAs are still worthy of further exploring.

## Conclusion

In the present study, we uncovered a novel carcinogenic lncRNA that is radically upregulated in CRC. Data in our study point out that RP11-757G1.5 is related with poor CRC prognosis and its silencing suppresses CRC cell proliferation, migration, and invasion in vitro and in vivo. Mechanically, we demonstrate that RP11-757G1.5 exerts its oncogenic function by sponging miR-139-5p, thus upregulating YAP1 expression. Jointly, our findings offer insights into the potential of RP11-757G1.5 as a prognostic biomarker and therapeutic target in CRC.

## Supplementary information

**Additional file 1 Figure S1**. LncRNA RP11-757G1.5 expression is upregulated in CRC tissues and is associated with poor prognosis, Related to Fig. 1. **A-B** Representative HE staining of different TNM stages and lymph node metastasis in serial sections of clinical samples and paired normal tissues harvested from CRC patiens. Scale bars, 100 μm.

**Additional file 2 Figure S4**. RP11-757G1.5 accelerates CRC cell migration and invasion in vitro, Related to Fig. 4. **A** Wound-healing assay was executed to investigate the horizontal migration ability with RP11-757G1.5 knockdown or overexpression in SW620 (High metastatic) and HT-29 (Low metastatic) cells, and relative gap distance was calculated and plotted on a histogram. **B** Migration and invasion assays were used to investigate the vertical migration and invasion abilities with RP11-757G1.5 knockdown or overexpression in SW620 and HT-29 cells cells, and the number of cells was calculated and plotted on a histogram. Scale bars, 10 μm. *p < 0.05, **p < 0.01, ***p < 0.001 by Student’s *t*-test. Data are representative of at least three independent experiments.

**Additional file 3 Figure S5**. RP11-757G1.5 sponges miR-139-5p and modulate YAP1 expression, Related to Fig. 5. **A** RT-qPCR was performed to determine levels of miRNAs (miR-139-5p, miR-4438, miR-92a-3p, miR-135b-3p, miR-203a-3p) in cells of HCT116 post-transfection by pcDNA3.1-757G1.5. **B** The sequences for WT and Mut forms of miR-139-5p were shown in Fig. 5b. miR-139-5p was highly enriched in the sample pulled down by biotinylated RP11-757G1.5-WT rather than RP11-757G1.5-Mut. *p < 0.05 by Student’s *t*-test versus Bio-NC (or NC). *n.s* represented no significance.

**Additional file 4 Figure S6**. RP11-757G1.5 exerts tumor-promoting function in CRC by regulating the miR-139-5p/YAP1 axis, Related to Fig. 6. **A-D** The decreased cell proliferation ability in miR-139-5p mimic transfected CRC cells were reversed by YAP1 overexpression. The increased cell proliferation ability in miR-139-5p inhibitor transfected CRC cells were restored by YAP1 knockdown. The cell proliferation ability was measured by a colony formation and Edu proliferation assay. Scale bars, 25 μm. **E-H** The decreased cell migration and invasion ability in miR-139-5p mimic transfected CRC cells were were reversed by YAP1 overexpression. The increased cell migration and invasion ability in miR-139-5p mimic transfected CRC cells were were restored by YAP1 knockdown. The cell migration and invasion were detected by wound healing and transwell assays. Scale bars, 25 μm. *p < 0.05, **p < 0.01, ***p < 0.001 by Student’s *t*-test. Data are representative of at least three independent experiments.

**Additional file 5.**

## Data Availability

At the reasonable request of the authors concerned, data supporting the research results can be obtained.

## Abbreviations

CRC: Colorectal cancer
lncRNA: Long non-coding RNA
ISH: In situ hybridization
RNA-FISH: fluorescence in situ hybridization of ribonucleic acid
RT-qPCR: Reverse transcription-quantitative polymerase chain reaction
ceRNA: competitive endogenous RNA
pcDNA3.1-RP11-757G1.5: pcDNA3.1- or pcDNA3.1-757G1.5
sh-RP11-757G1.5#1, sh-RP11-757G1.5#2: sh-757G1.5#1, sh-757G1.5#2
YAP1: Yes-associated protein 1
IHC: Immunohistochemistry
RIP: RNA Immunoprecipitation
