# Supplementary figures and images for: Long noncoding RNA RP11-757G1.5 sponges miR-139-5p and upregulates YAP1 thereby promoting the proliferation and liver, spleen metastasis of colorectal cancer
Source: J Exp Clin Cancer Res. 2020 Oct 6;39:207. doi: 10.1186/s13046-020-01717-5 (PMC7541316; doi:10.1186/s13046-020-01717-5)

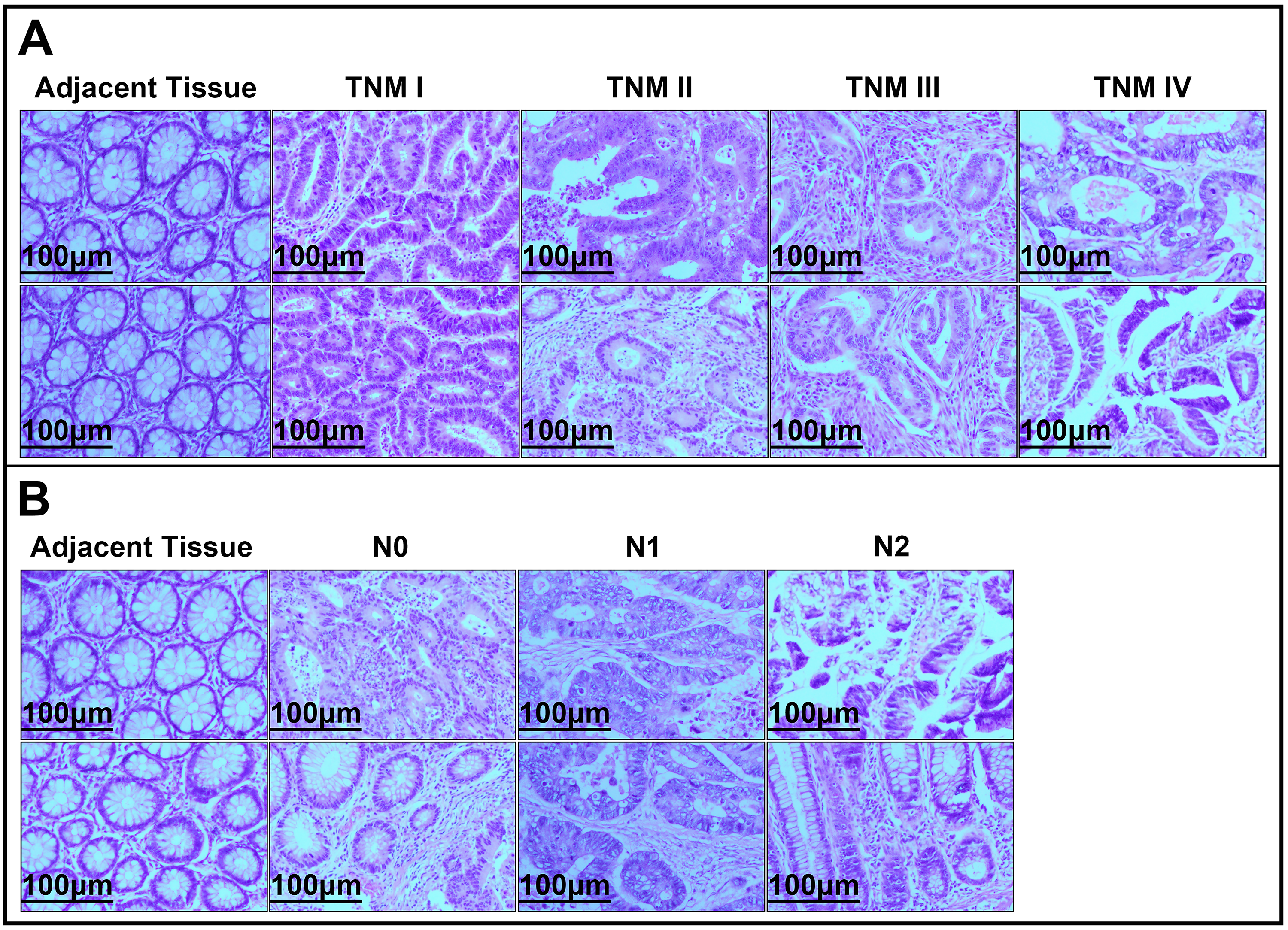

Supplement: Supplementary file 1 — Additional file 1 Figure S1. LncRNA RP11-757G1.5 expression is upregulated in CRC tissues and is associated with poor prognosis, Related to Fig. 1. A-B Representative HE staining of different TNM stages and lymph node metastasis in serial sections of clinical samples and paired normal tissues harvested from CRC patiens. Scale bars, 100 μm. [file 13046_2020_1717_MOESM1_ESM.tif]

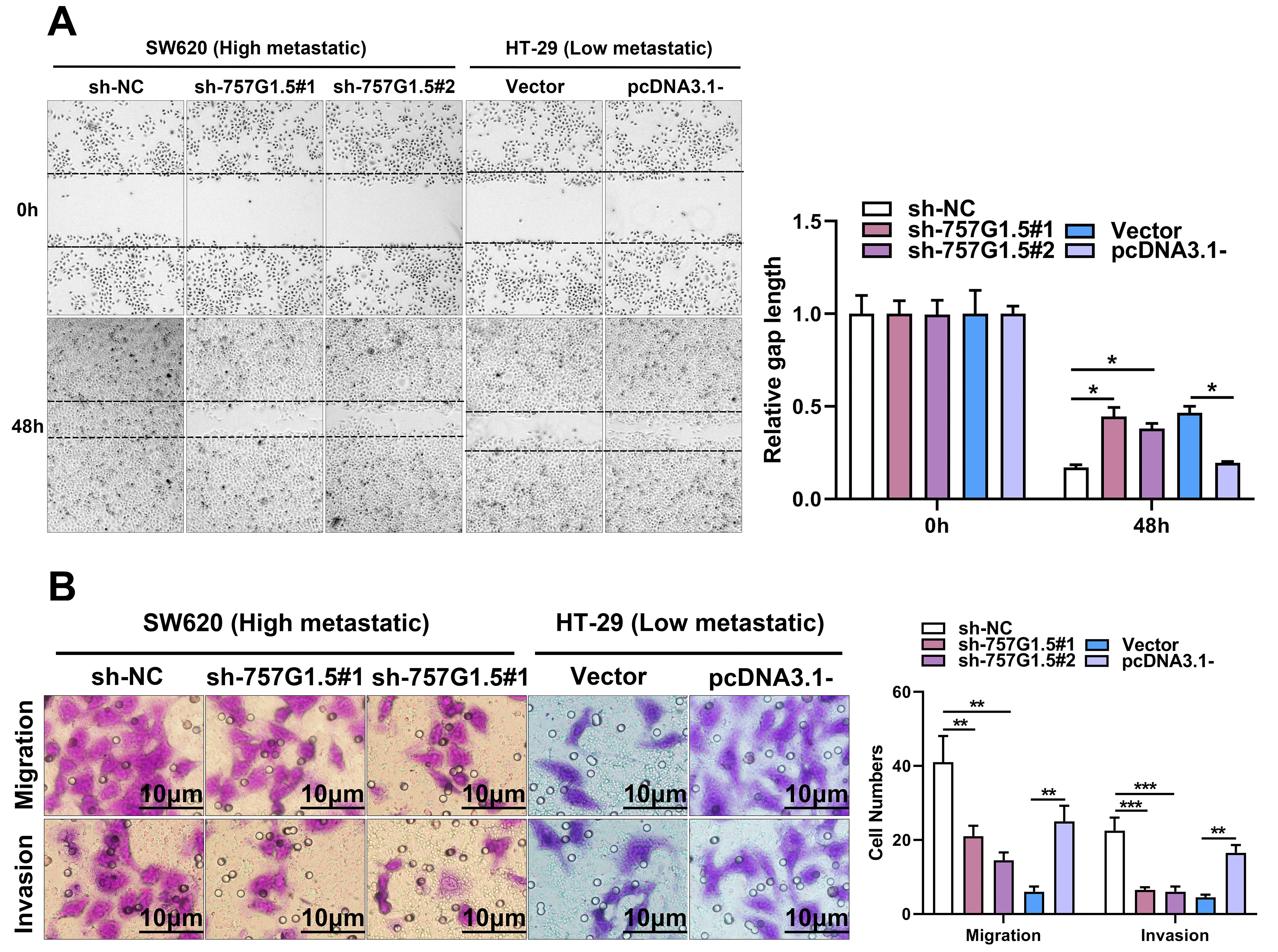

Supplement: Supplementary file 2 — Additional file 2 Figure S4. RP11-757G1.5 accelerates CRC cell migration and invasion in vitro, Related to Fig. 4. A Wound-healing assay was executed to investigate the horizontal migration ability with RP11-757G1.5 knockdown or overexpression in SW620 (High metastatic) and HT-29 (Low metastatic) cells, and relative gap distance was calculated and plotted on a histogram. B Migration and invasion assays were used to investigate the vertical migration and invasion abilities with RP11-757G1.5 knockdown or overexpression in SW620 and HT-29 cells cells, and the number of cells was calculated and plotted on a histogram. Scale bars, 10 μm. *p < 0.05, **p < 0.01, ***p < 0.001 by Student’s t-test. Data are representative of at least three independent experiments. [file 13046_2020_1717_MOESM2_ESM.tif]

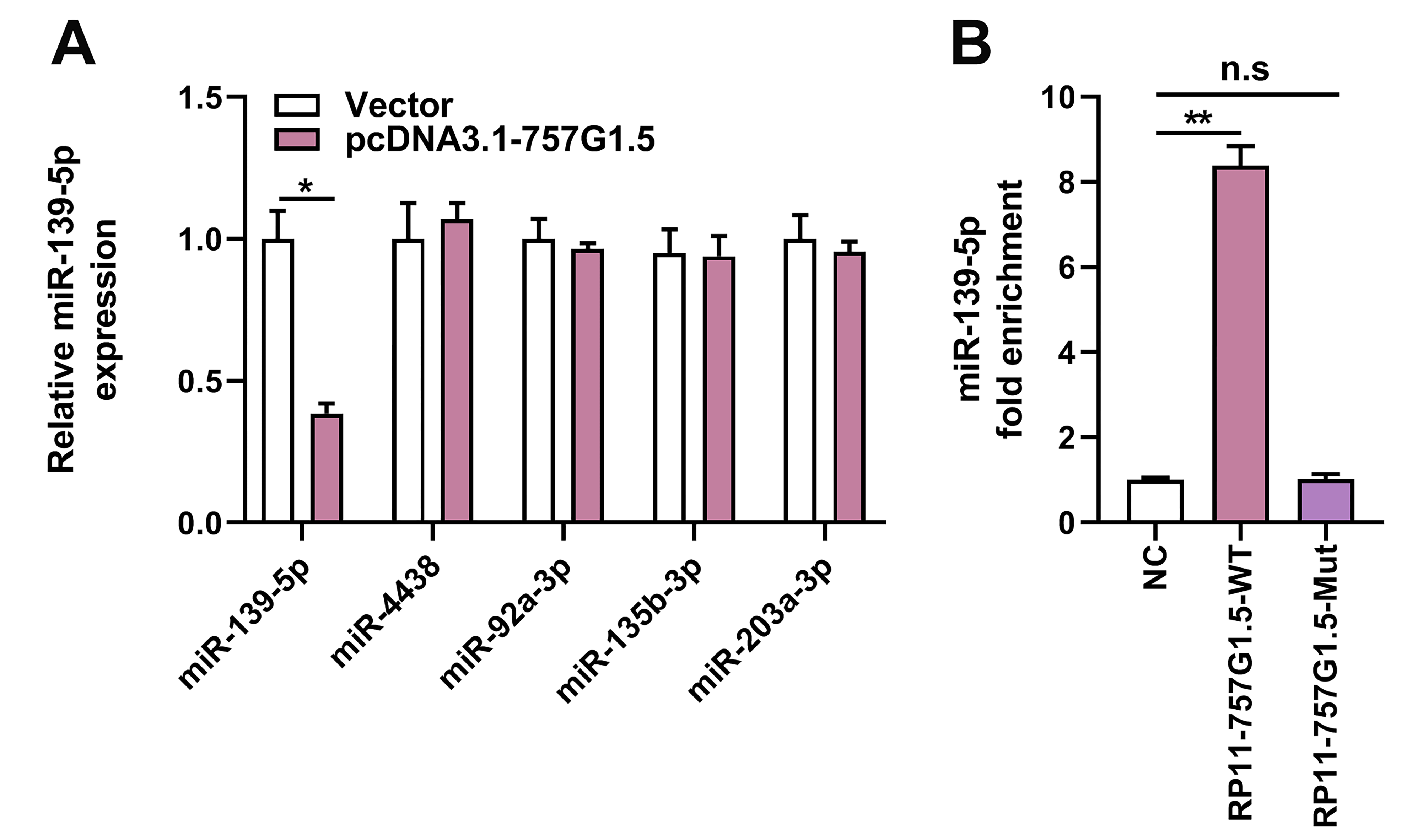

Supplement: Supplementary file 3 — Additional file 3 Figure S5. RP11-757G1.5 sponges miR-139-5p and modulate YAP1 expression, Related to Fig. 5. A RT-qPCR was performed to determine levels of miRNAs (miR-139-5p, miR-4438, miR-92a-3p, miR-135b-3p, miR-203a-3p) in cells of HCT116 post-transfection by pcDNA3.1-757G1.5. B The sequences for WT and Mut forms of miR-139-5p were shown in Fig. 5b. miR-139-5p was highly enriched in the sample pulled down by biotinylated RP11-757G1.5-WT rather than RP11-757G1.5-Mut. *p < 0.05 by Student’s t-test versus Bio-NC (or NC). n.s represented no significance. [file 13046_2020_1717_MOESM3_ESM.tif]

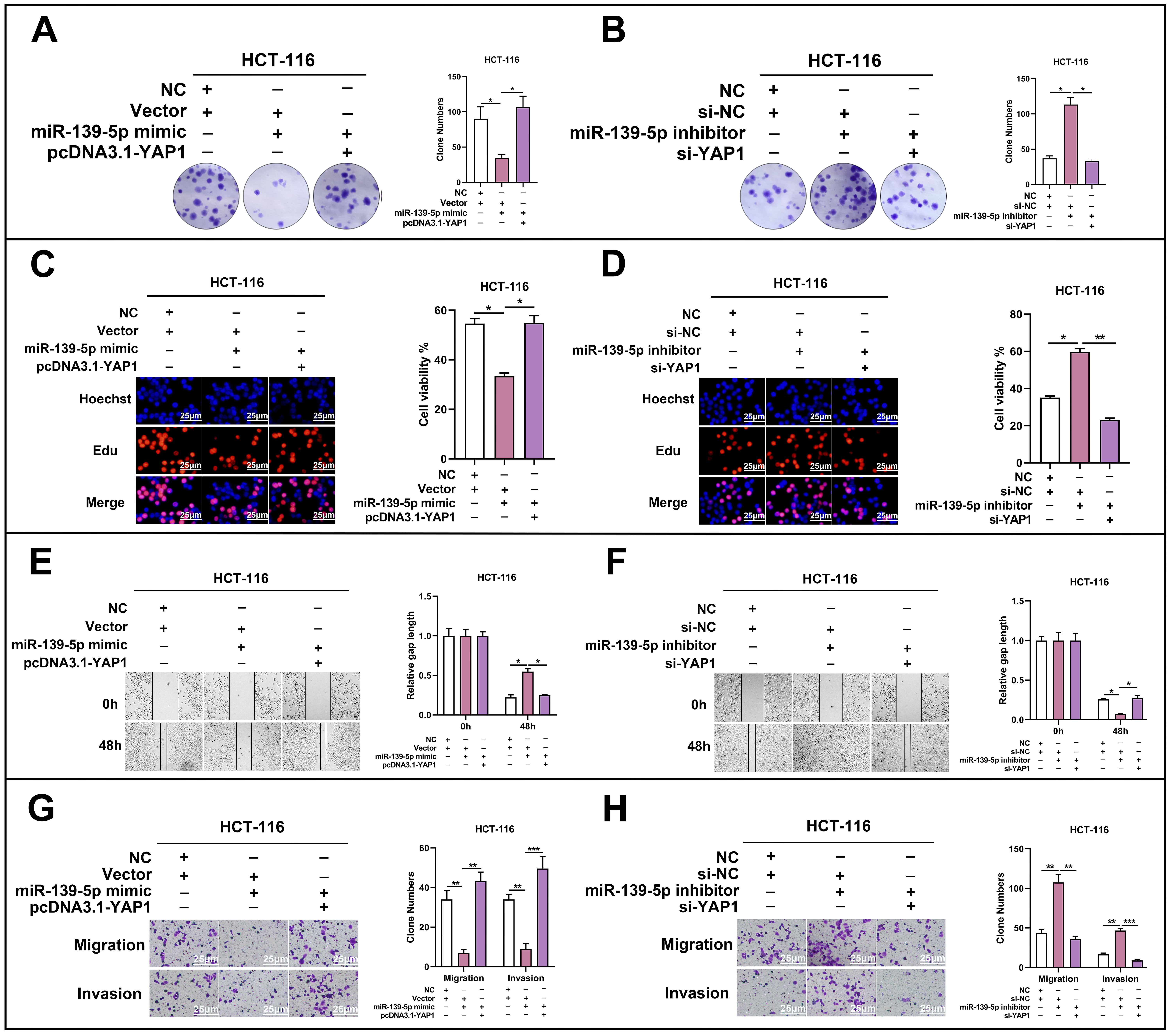

Supplement: Supplementary file 4 — Additional file 4 Figure S6. RP11-757G1.5 exerts tumor-promoting function in CRC by regulating the miR-139-5p/YAP1 axis, Related to Fig. 6. A-D The decreased cell proliferation ability in miR-139-5p mimic transfected CRC cells were reversed by YAP1 overexpression. The increased cell proliferation ability in miR-139-5p inhibitor transfected CRC cells were restored by YAP1 knockdown. The cell proliferation ability was measured by a colony formation and Edu proliferation assay. Scale bars, 25 μm. E-H The decreased cell migration and invasion ability in miR-139-5p mimic transfected CRC cells were were reversed by YAP1 overexpression. The increased cell migration and invasion ability in miR-139-5p mimic transfected CRC cells were were restored by YAP1 knockdown. The cell migration and invasion were detected by wound healing and transwell assays. Scale bars, 25 μm. *p < 0.05, **p < 0.01, ***p < 0.001 by Student’s t-test. Data are representative of at least three independent experiments. [file 13046_2020_1717_MOESM4_ESM.tif]
