# Supplementary material for: Long noncoding RNA RP11-757G1.5 sponges miR-139-5p and upregulates YAP1 thereby promoting the proliferation and liver, spleen metastasis of colorectal cancer
Source: J Exp Clin Cancer Res. 2020 Oct 6;39:207. doi: 10.1186/s13046-020-01717-5 (PMC7541316; doi:10.1186/s13046-020-01717-5)
Supplement: Supplementary file 5 — Additional file 5. [file 13046_2020_1717_MOESM5_ESM.pptx]

## Slide 1
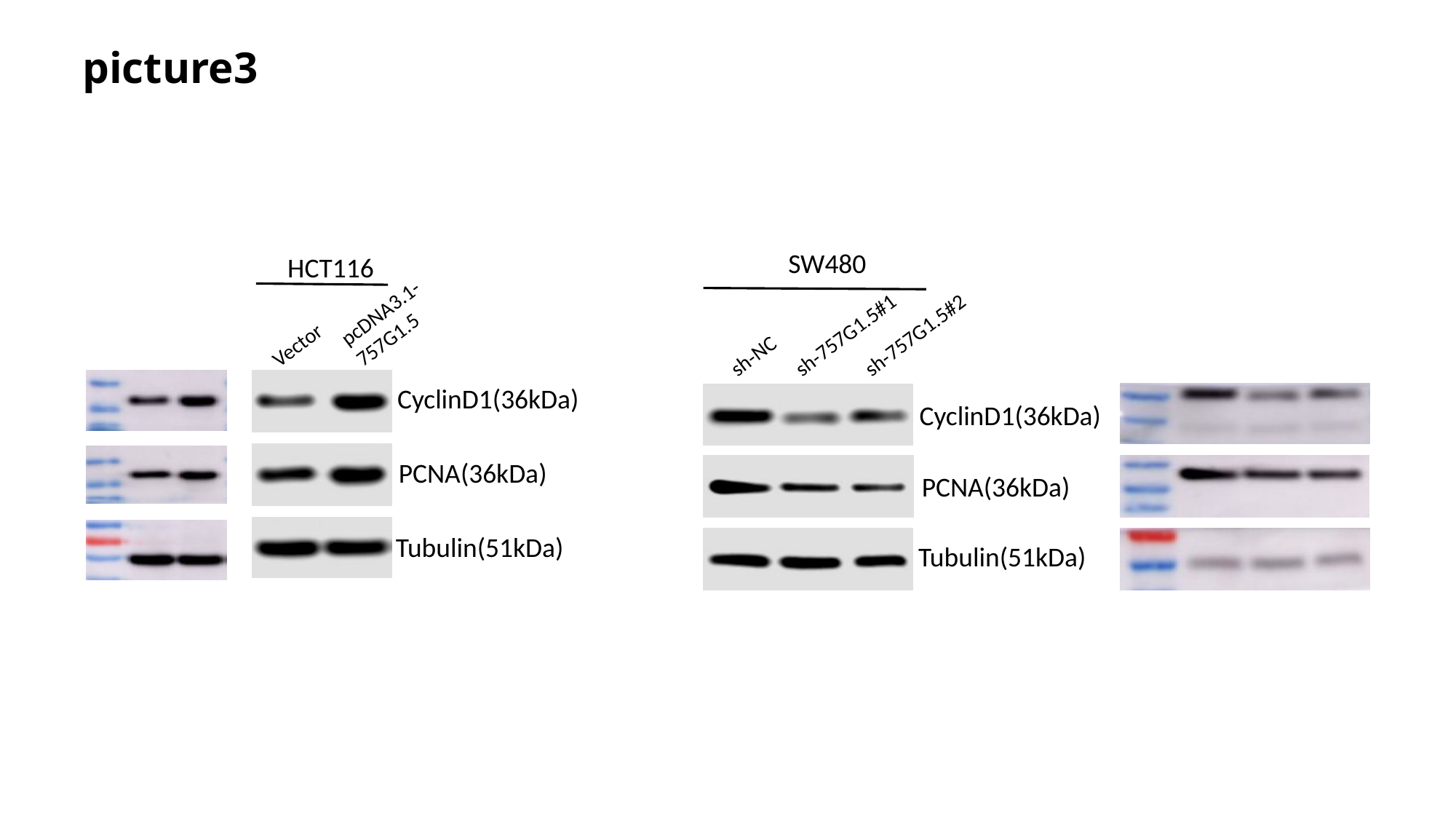

picture3
SW480
sh-757G1.5#1
sh-757G1.5#2
sh-NC
CyclinD1(36kDa)
PCNA(36kDa)
Tubulin(51kDa)
HCT116
pcDNA3.1-757G1.5
Vector
CyclinD1(36kDa)
PCNA(36kDa)
Tubulin(51kDa)

## Slide 2
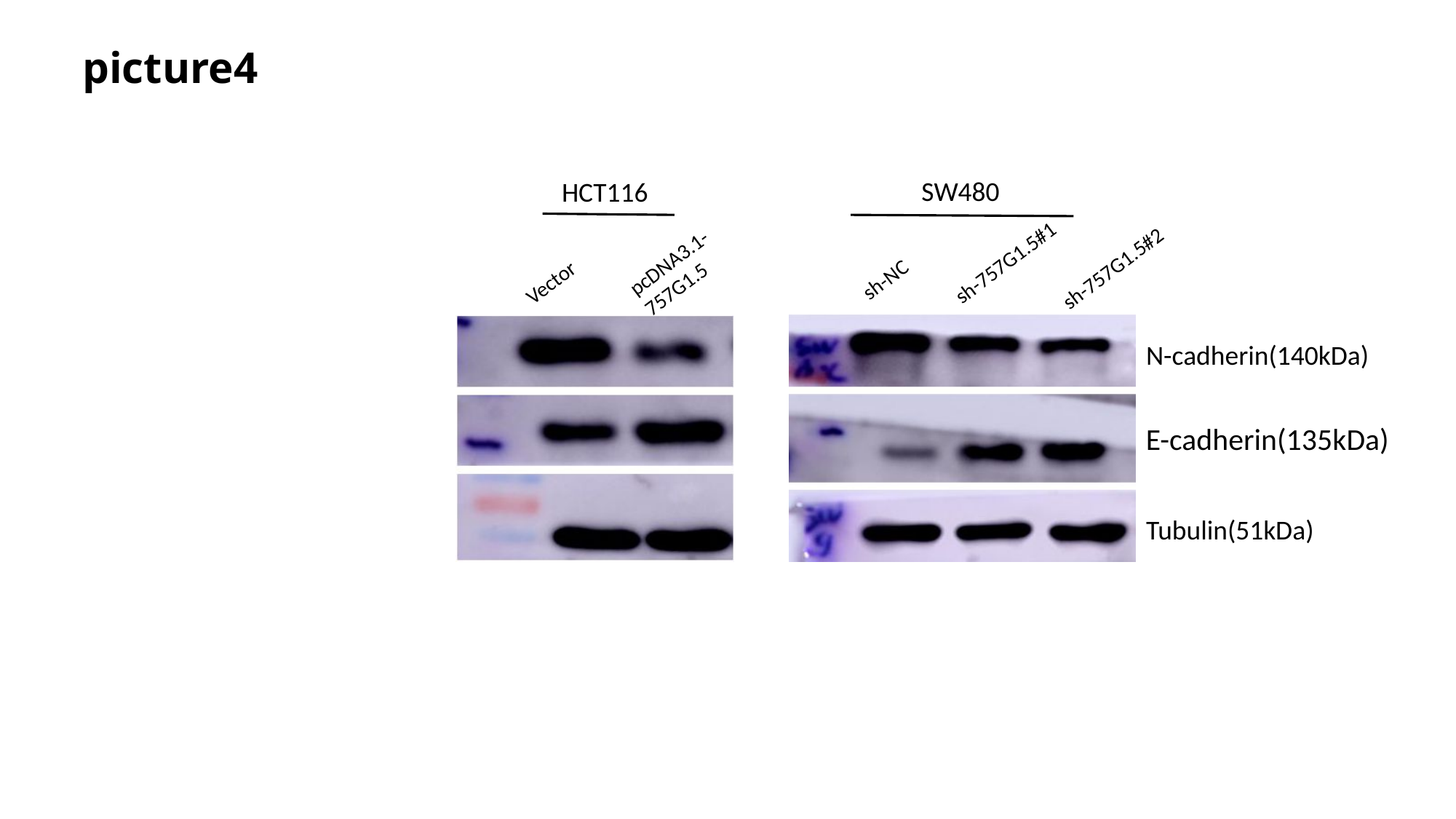

picture4
SW480
HCT116
pcDNA3.1-757G1.5
sh-757G1.5#1
sh-757G1.5#2
sh-NC
Vector
N-cadherin(140kDa)
E-cadherin(135kDa)
Tubulin(51kDa)

## Slide 3
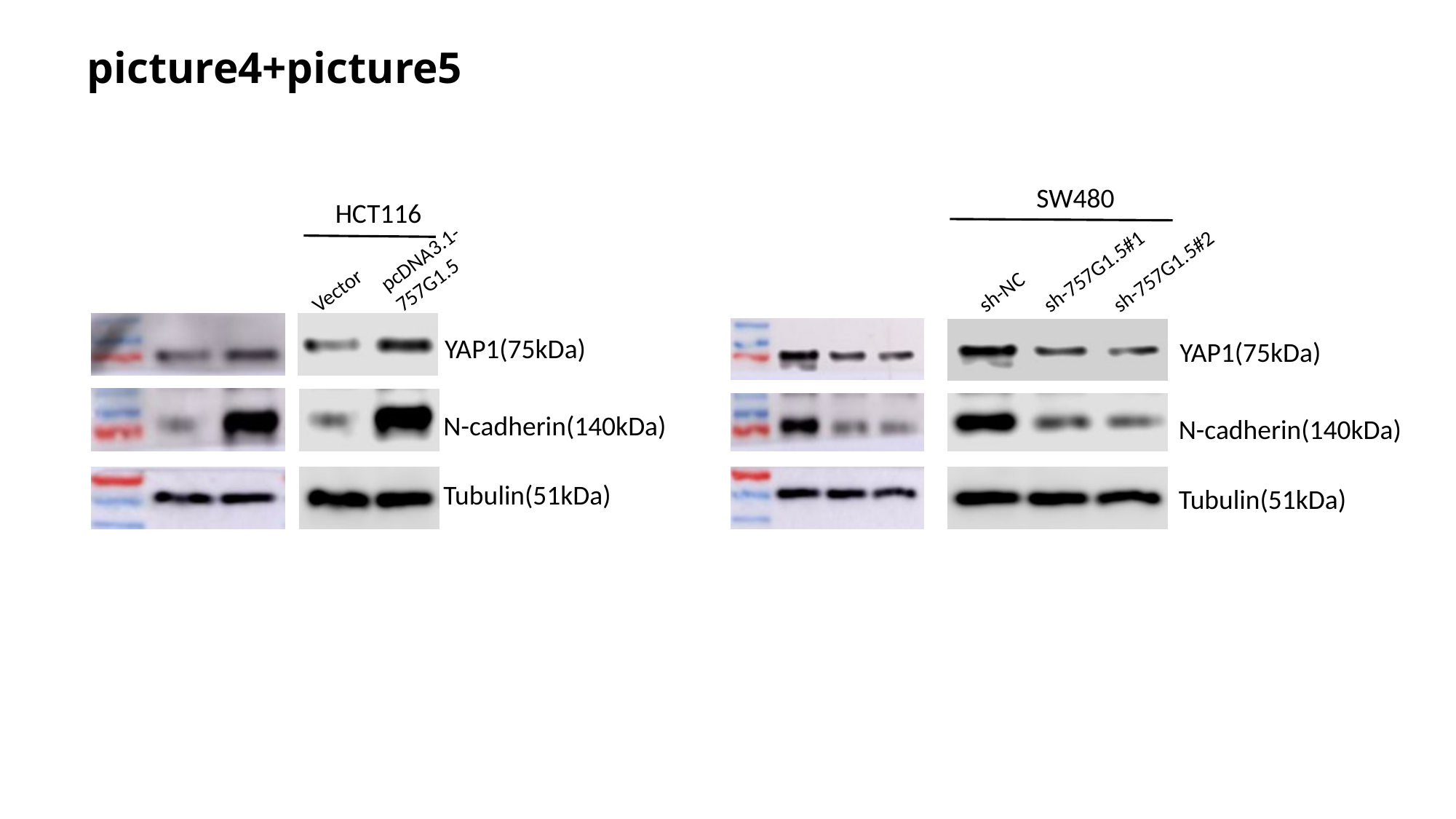

picture4+picture5
SW480
HCT116
pcDNA3.1-757G1.5
Vector
sh-757G1.5#1
sh-757G1.5#2
sh-NC
YAP1(75kDa)
N-cadherin(140kDa)
Tubulin(51kDa)
YAP1(75kDa)
N-cadherin(140kDa)
Tubulin(51kDa)

## Slide 4
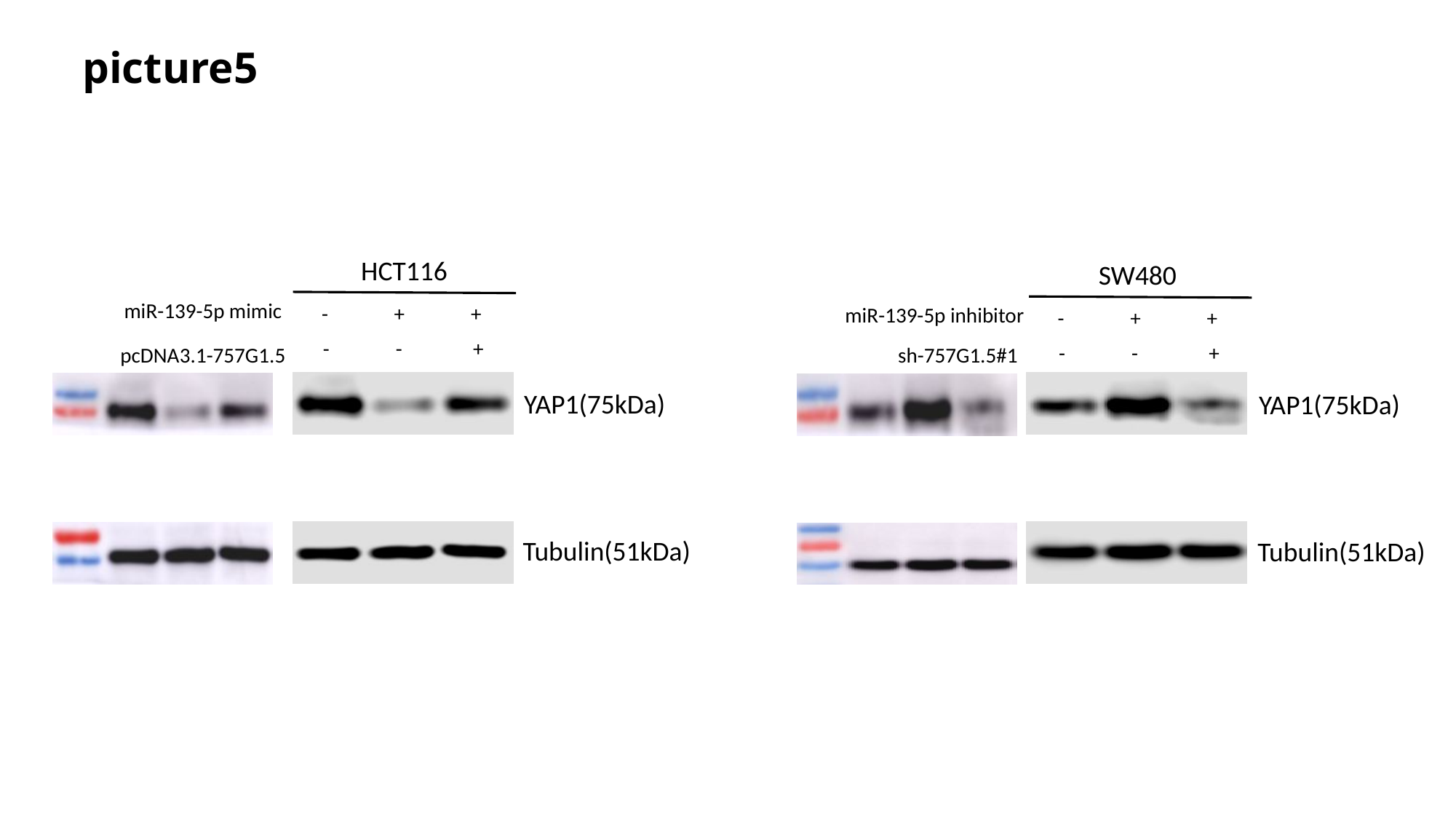

picture5
HCT116
SW480
miR-139-5p mimic
- + +
miR-139-5p inhibitor
- + +
- - +
- - +
pcDNA3.1-757G1.5
sh-757G1.5#1
YAP1(75kDa)
Tubulin(51kDa)
YAP1(75kDa)
Tubulin(51kDa)

## Slide 5
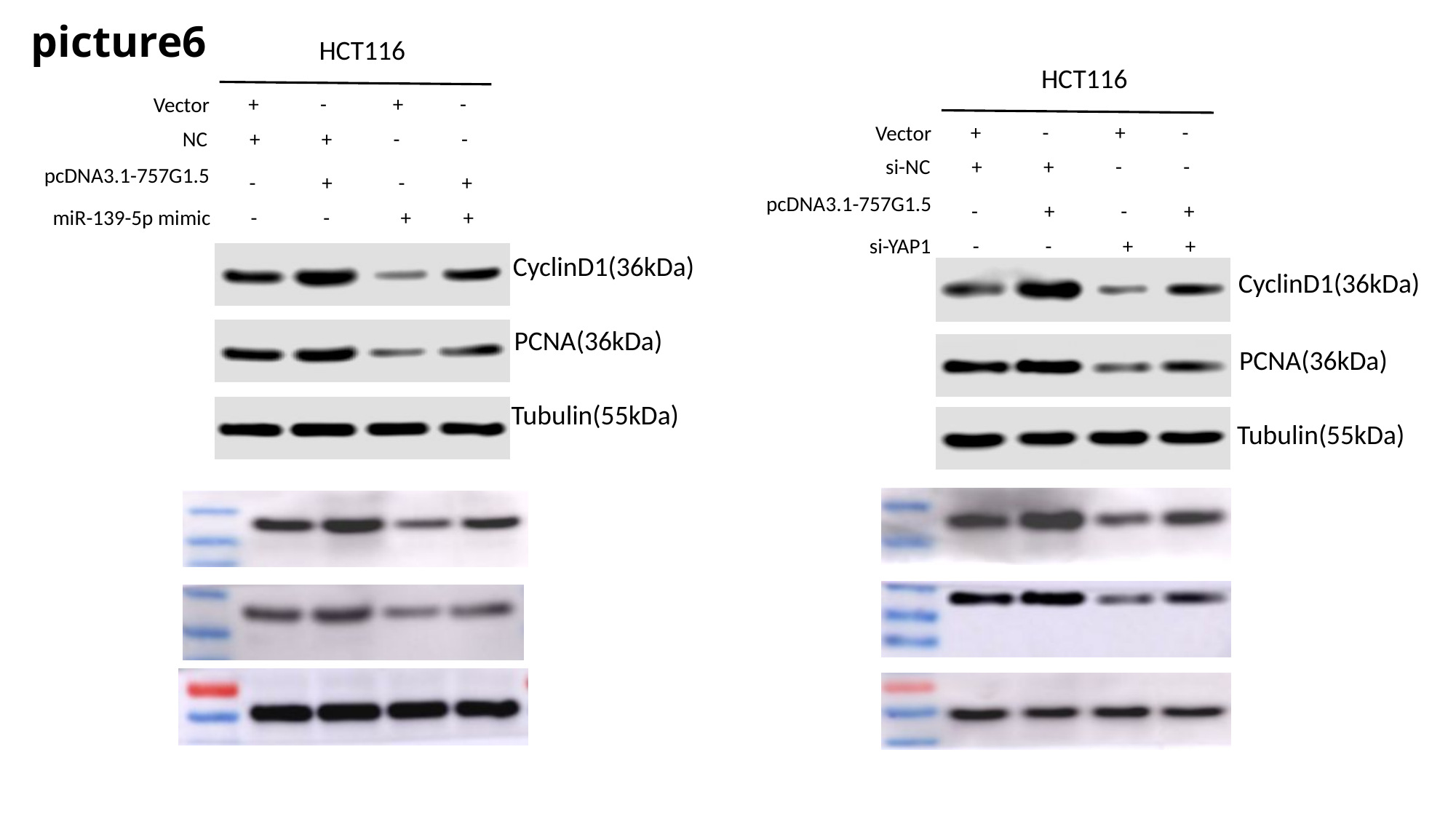

picture6
HCT116
+ - + -
Vector
 NC
+ + - -
pcDNA3.1-757G1.5
- + - +
miR-139-5p mimic
- - + +
CyclinD1(36kDa)
PCNA(36kDa)
Tubulin(55kDa)
HCT116
+ - + -
Vector
si-NC
+ + - -
pcDNA3.1-757G1.5
- + - +
si-YAP1
- - + +
CyclinD1(36kDa)
PCNA(36kDa)
Tubulin(55kDa)
